# Supplementary material for: Sex and neo-sex chromosome evolution in beetles
Source: PLoS Genet. 2024 Nov 25;20(11):e1011477. doi: 10.1371/journal.pgen.1011477 (PMC11753715; doi:10.1371/journal.pgen.1011477)
Supplement: S1 Table — (PDF) [file pgen.1011477.s001.pdf]

**Supplemental Table 1.** BUSCO results from each round of assembly and polishing of the draft genome for *Tribolium confusum*.

| | Canu/WTDBG2 only | Canu/WTDBG2 + Racon 3x | Canu/WTDBG2 + Racon 3x + Pilon 3x | Final Hi-C scaffolded Tcon_1.0 |
|---|---|---|---|---|
| Complete BUSCOs | 922 | 992 | 1043 | 1043 |
| Single-copy BUSCOs | 902 | 967 | 1017 | 1017 |
| Duplicated BUSCOs | 20 | 25 | 26 | 26 |
| Fragmented BUSCOs | 86 | 48 | 6 | 6 |
| Missing BUSCOs | 58 | 26 | 17 | 17 |
| | | | | |
| % BUSCOs complete | 86.5% | 93.1% | 97.8% | 97.8% |
