# Supplementary material for: Sex and neo-sex chromosome evolution in beetles
Source: PLoS Genet. 2024 Nov 25;20(11):e1011477. doi: 10.1371/journal.pgen.1011477 (PMC11753715; doi:10.1371/journal.pgen.1011477)
Supplement: S2 Table — (PDF) [file pgen.1011477.s002.pdf]

Supplemental Table 2. Description of the eleven genomes used in comparative analyses.

species	assembly version	male karyotype	Assembly length (bp)	1:1 orthologs with Tcas	X-linked	X-linked inTcas
Ldec	2.0	17AA + XO	641,992,784	7,922	168	146
Apla	2.0	?	353,615,807	7,259	392	316
Agla	2.0	9AA + XY	706,968,555	8,315	589	456
Nves	1.0	?	195,273,382	8,206	525	457
Pjap	here	9AA + XY	851,227,647	5,247	421	378
Pcha	1.0	10AA + XY	280,876,046	6,382	104	86
Ppyr	1.3	9AA + XO	471,511,253	6,932	433	350
Dpon	Dpon_F_20191213v2	11AA + neoXY	223,739,972	6,283	1,513	344
Tcas	5.2	9AA + XY	165,944,485	-	-	-
Tcon	here	8AA + neoXY	305,133,470	10,138	629	625
Caen	1.0	?	354,059,554	7,677	454	385
