# Supplementary material for: Sex and neo-sex chromosome evolution in beetles
Source: PLoS Genet. 2024 Nov 25;20(11):e1011477. doi: 10.1371/journal.pgen.1011477 (PMC11753715; doi:10.1371/journal.pgen.1011477)
Supplement: S1 Fig — Mean female (top) and male (bottom) Nanopore sequencing coverage (log2) plotted along the Tribolium confusum genome assembly in 50 kb windows. (PDF) [file pgen.1011477.s003.pdf]

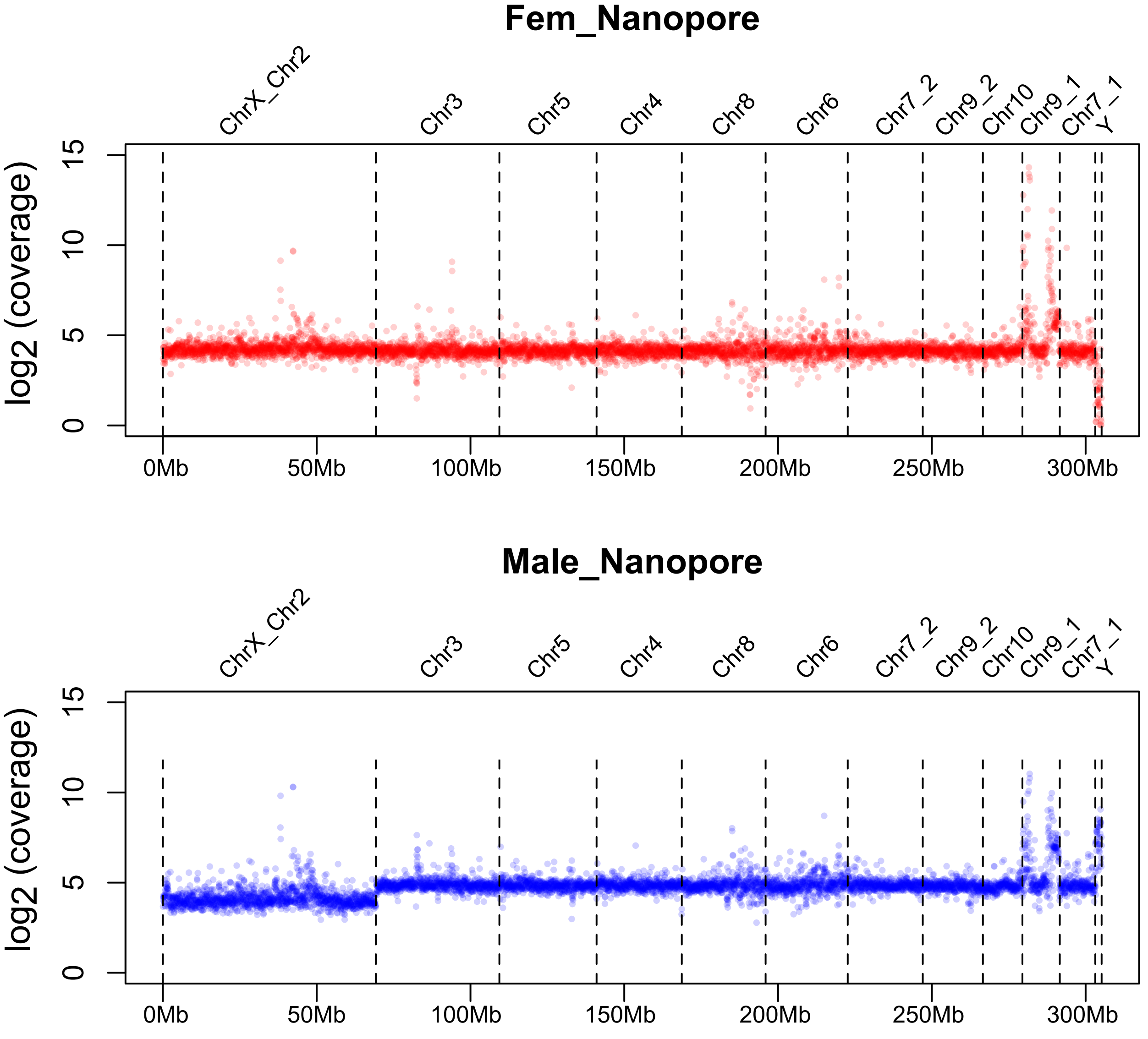

Supplemental Figure 1. Mean female (top) and male (bottom) Nanopore sequencing coverage (log2) plotted along the *Tribolium confusum* genome assembly in 50kb windows.
