# Supplementary material for: Sex and neo-sex chromosome evolution in beetles
Source: PLoS Genet. 2024 Nov 25;20(11):e1011477. doi: 10.1371/journal.pgen.1011477 (PMC11753715; doi:10.1371/journal.pgen.1011477)
Supplement: S2 Fig — (PDF) [file pgen.1011477.s004.pdf]

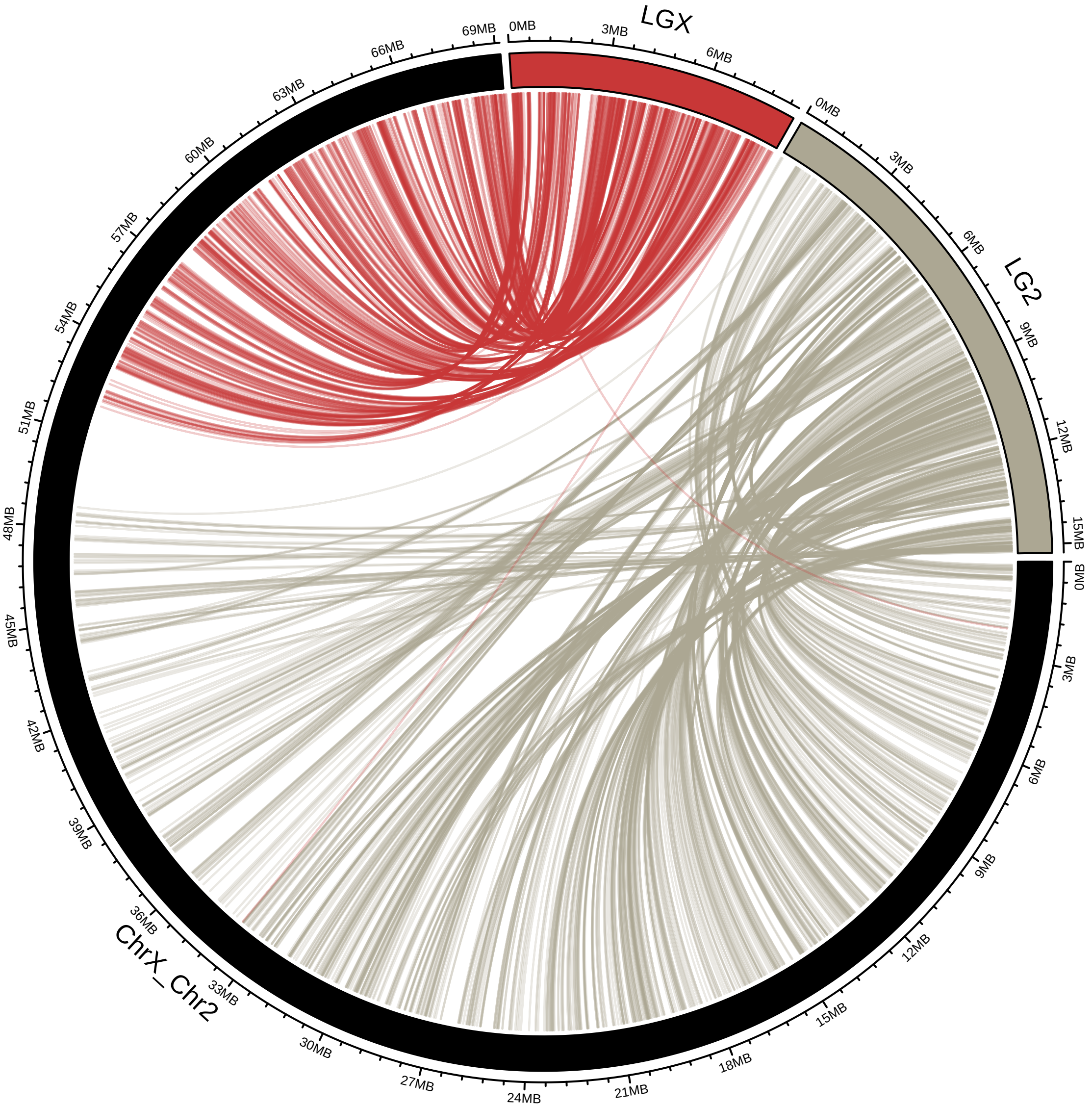

Supplemental Figure 2. 1,621 1:1 orthologs of *Tribolium castaneum* LGX and LG2 and their relative locations on the fused *Tribolium confusum* ChrX_Chr2 chromosome.
