# Supplementary material for: Sex and neo-sex chromosome evolution in beetles
Source: PLoS Genet. 2024 Nov 25;20(11):e1011477. doi: 10.1371/journal.pgen.1011477 (PMC11753715; doi:10.1371/journal.pgen.1011477)
Supplement: S3 Fig — Putative Stevens elements are shown on the horizontal (A-H, X) while species-specific naming schemes for linkage groups from each draft genome assembly are shown on the vertical. Note that LG10 in Tribolium castaneum (salmon) is shown but was not found to be conserved over time and is not considered a Stevens element. (PDF) [file pgen.1011477.s005.pdf]

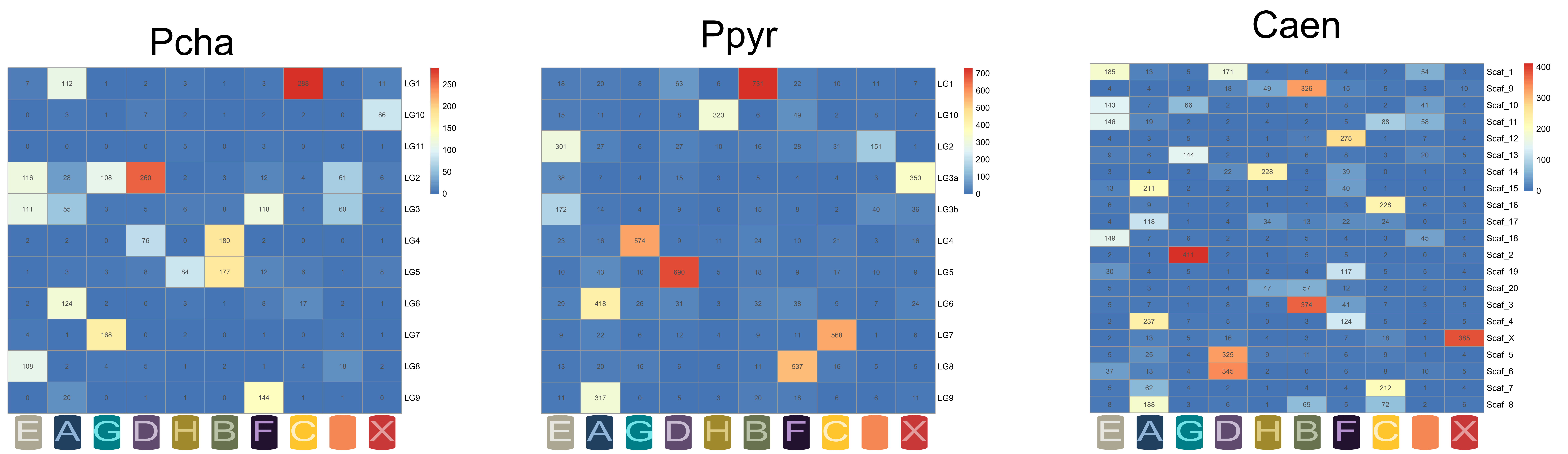

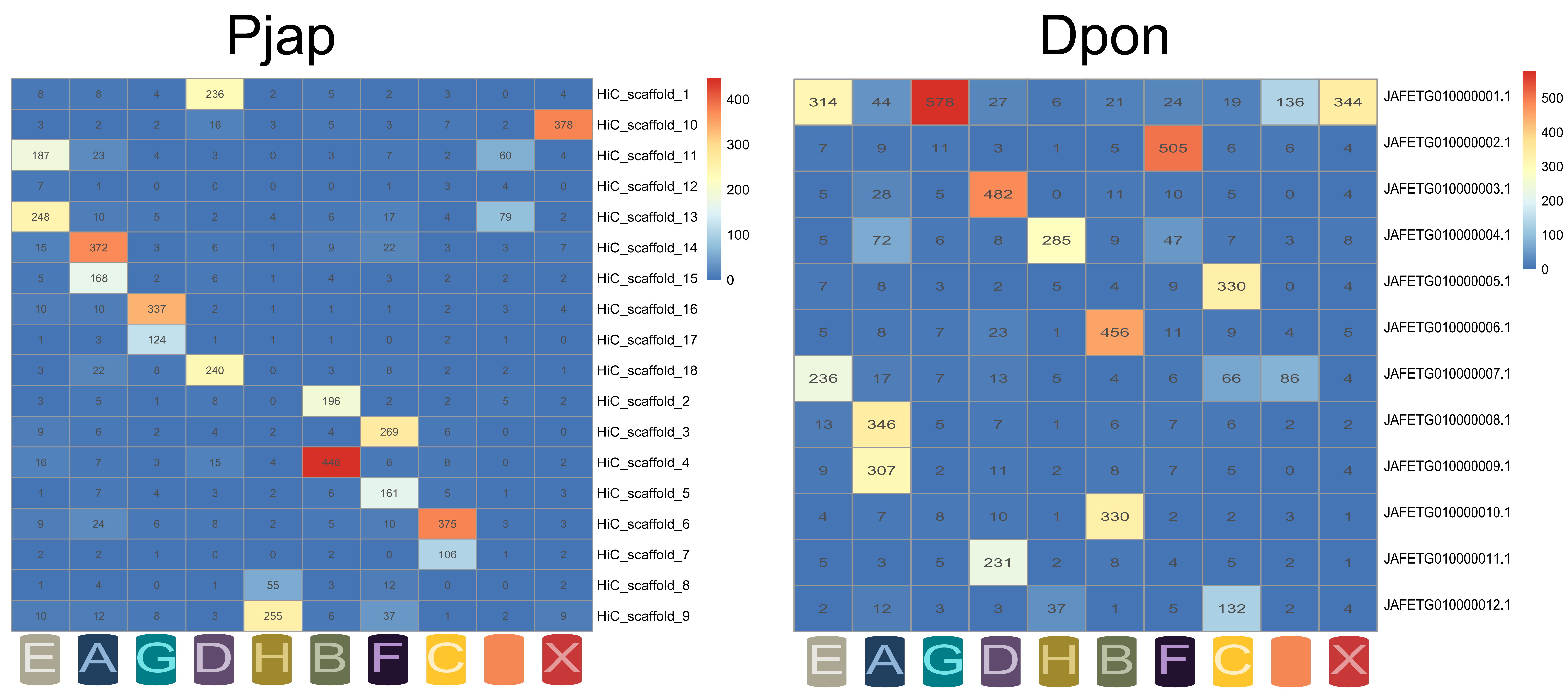

Supplemental Figure 3. Heatmaps showing locations of 1:1 orthologs in genome comparisons with other beetle species with chromosome-level assemblies. Putative Stevens elements are shown on the horizontal (A-H, X) while species-specific naming schemes for linkage groups from each draft genome assembly are shown on the vertical. Note that LG10 in *Tribolium castaneum* (salmon) is shown but was not found to be conserved over time and is not considered a Stevens element.
