# Supplementary material for: Sex and neo-sex chromosome evolution in beetles
Source: PLoS Genet. 2024 Nov 25;20(11):e1011477. doi: 10.1371/journal.pgen.1011477 (PMC11753715; doi:10.1371/journal.pgen.1011477)
Supplement: S4 Fig — Synteny across five species from the suborder Polyphaga using 2,244 1:1 orthologs color coded by their chromosomal placement in Tribolium castaneum. Stevens elements correspond to conserved linkage groups of the genome in beetles. (PDF) [file pgen.1011477.s006.pdf]

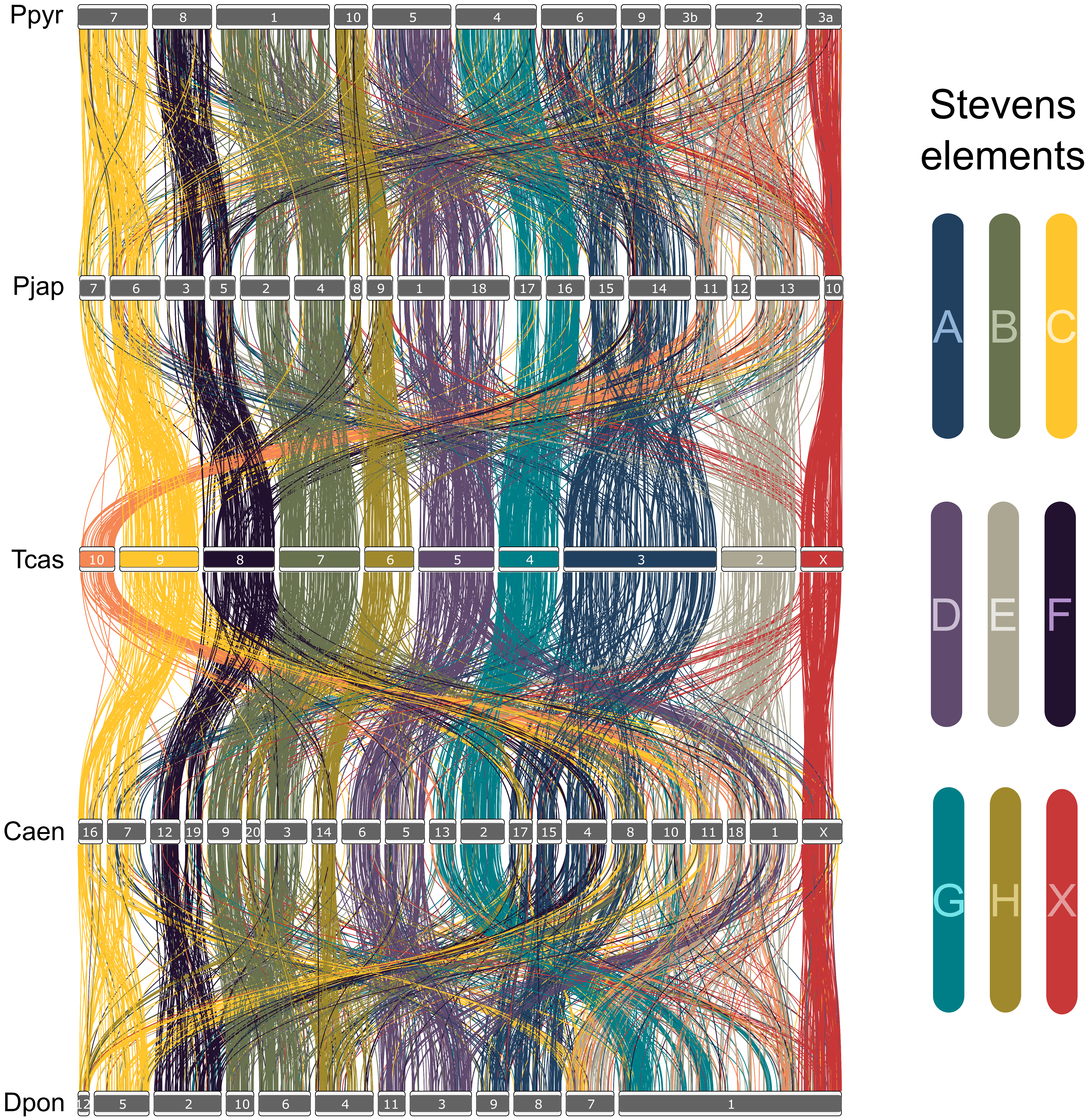

Supplemental Figure 4. Chromosome conservation in beetles. Synteny across five species from the suborder Polyphaga using 2,244 1:1 orthologs color coded by their chromosomal placement in *Tribolium castaneum*. Stevens elements correspond to conserved linkage groups of the genome in beetles.
