# Supplementary material for: Sex and neo-sex chromosome evolution in beetles
Source: PLoS Genet. 2024 Nov 25;20(11):e1011477. doi: 10.1371/journal.pgen.1011477 (PMC11753715; doi:10.1371/journal.pgen.1011477)
Supplement: S5 Fig — A) Hi-C contact map showing the published scaffolded Propylaea japonica genome assembly (Linkage groups 0–9 from left to right). We found widespread within-linkage group scaffolding that was not supported by the Hi-C data. B) Our re-scaffolded assembly with linkage groups shown on the vertical (right). Our naming scheme follows the original LG name from the published assembly followed by additional notation when we were unable to confidently orient into one linkage group. For example, LG1_1 and LG1_2 represent two scaffolds that originate from the published LG1. LG1_1 and LG1_2 likely represent two arms of a single chromosome. (PDF) [file pgen.1011477.s007.pdf]

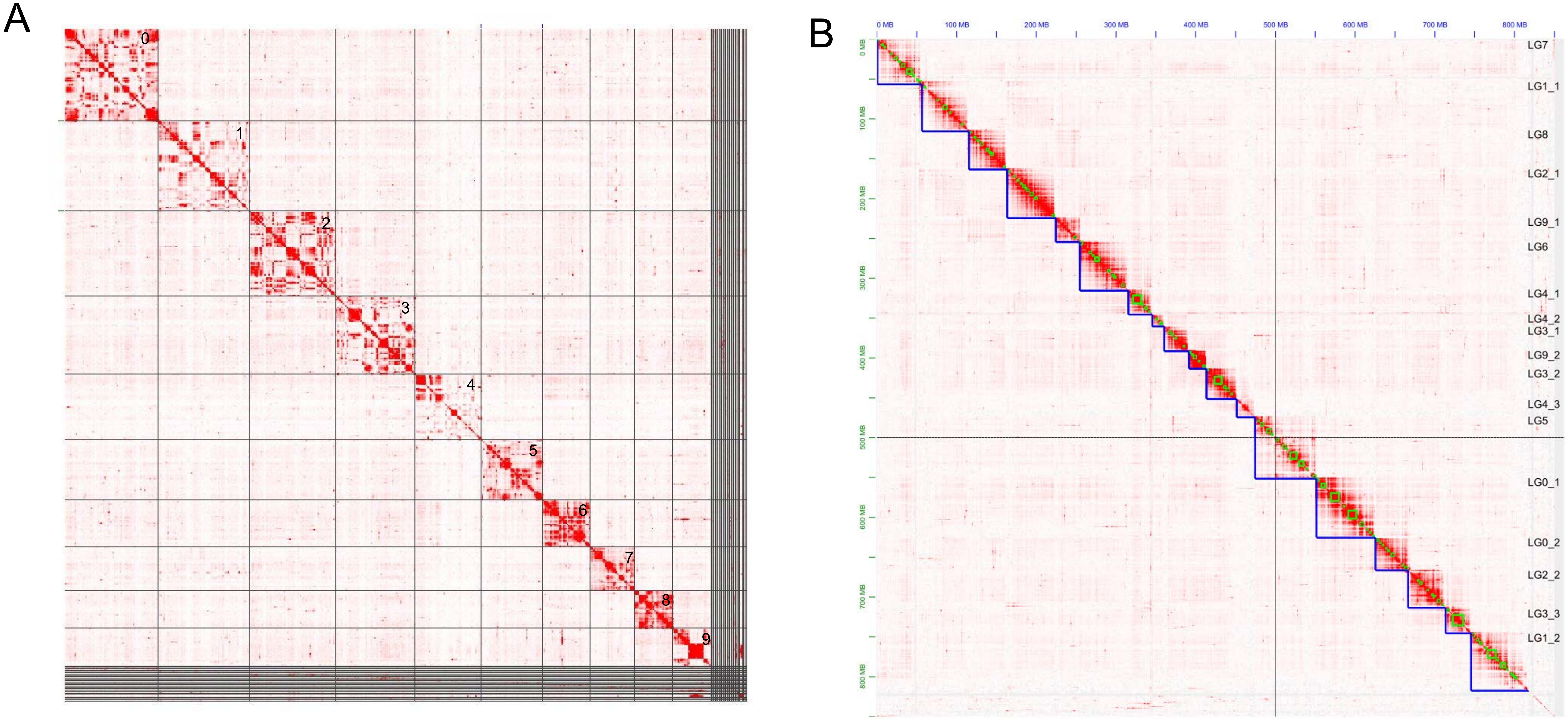

Supplemental Figure 5. A) Hi-C contact map showing the published scaffolded *Propylaea japonica* genome assembly (Linkage groups 0 - 9 from left to right). We found widespread within-linkage group scaffolding that was not supported by the Hi-C data. B) Our re-scaffolded assembly with linkage groups shown on the vertical (right). Our naming scheme follows the original LG name from the published assembly followed by additional notation when we were unable to confidently orient into one linkage group. For example, LG1_1 and LG1_2 represent two scaffolds that originate from the published LG1. LG1_1 and LG1_2 likely represent two arms of a single chromosome.
