# Supplementary figures and images for: Sex and neo-sex chromosome evolution in beetles
Source: PLoS Genet. 2024 Nov 25;20(11):e1011477. doi: 10.1371/journal.pgen.1011477 (PMC11753715; doi:10.1371/journal.pgen.1011477)

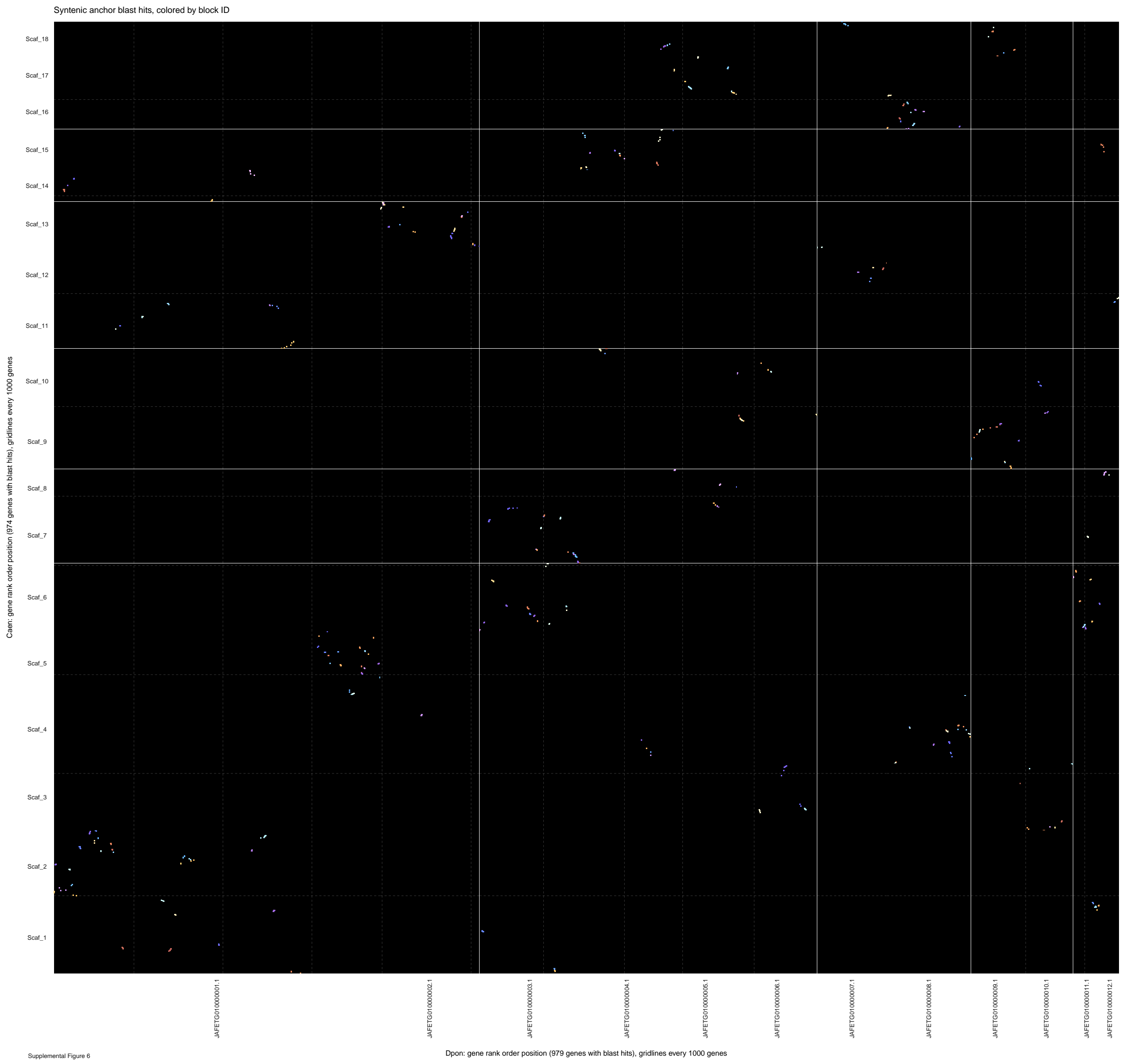

Supplement: S6 Fig — (PDF) [file pgen.1011477.s008.pdf]

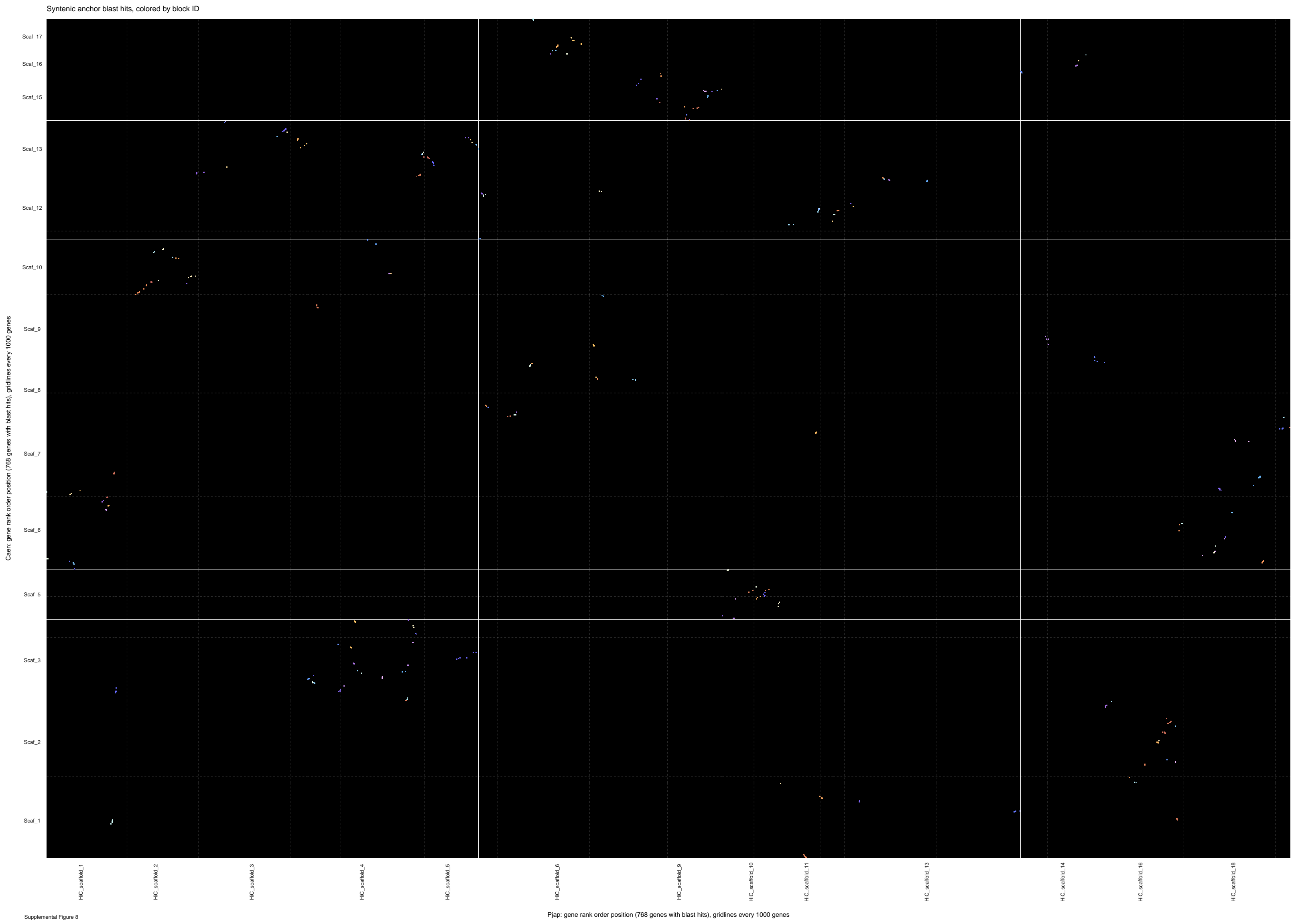

Supplement: S8 Fig — (PDF) [file pgen.1011477.s010.pdf]

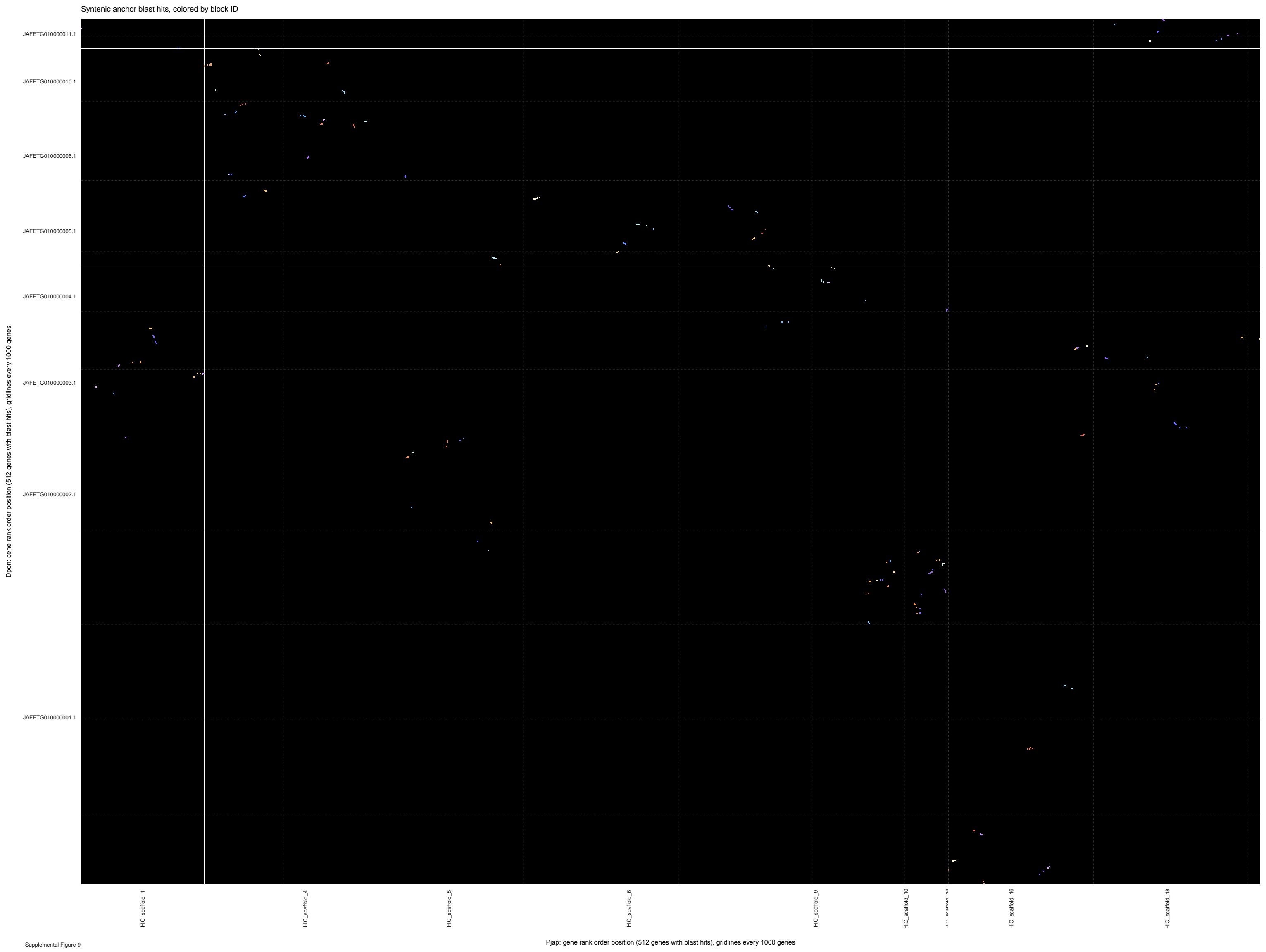

Supplement: S9 Fig — (PDF) [file pgen.1011477.s011.pdf]

Syntenic anchor blast hits, colored by block ID

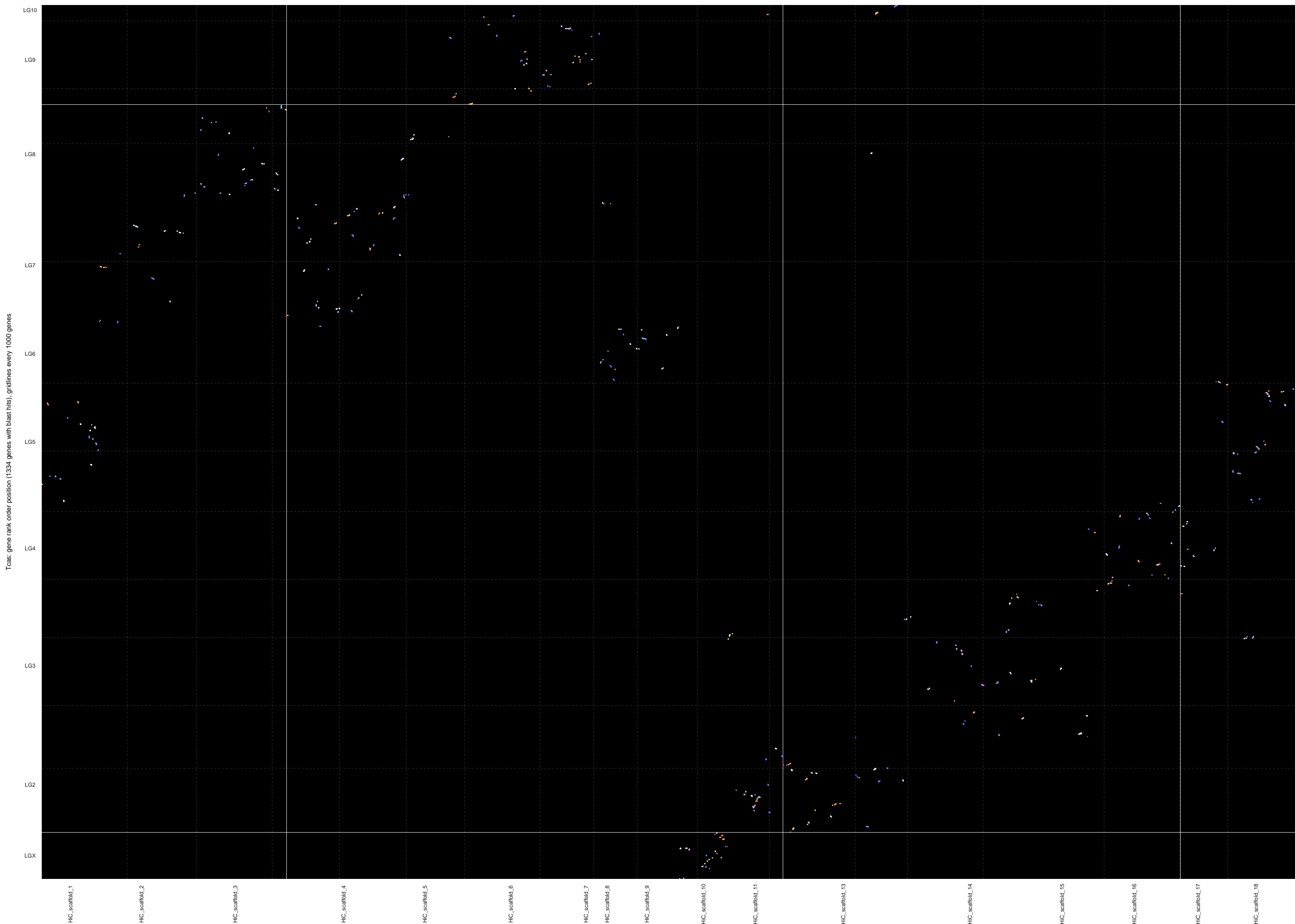

Supplement: S11 Fig — (PDF) [file pgen.1011477.s013.pdf]

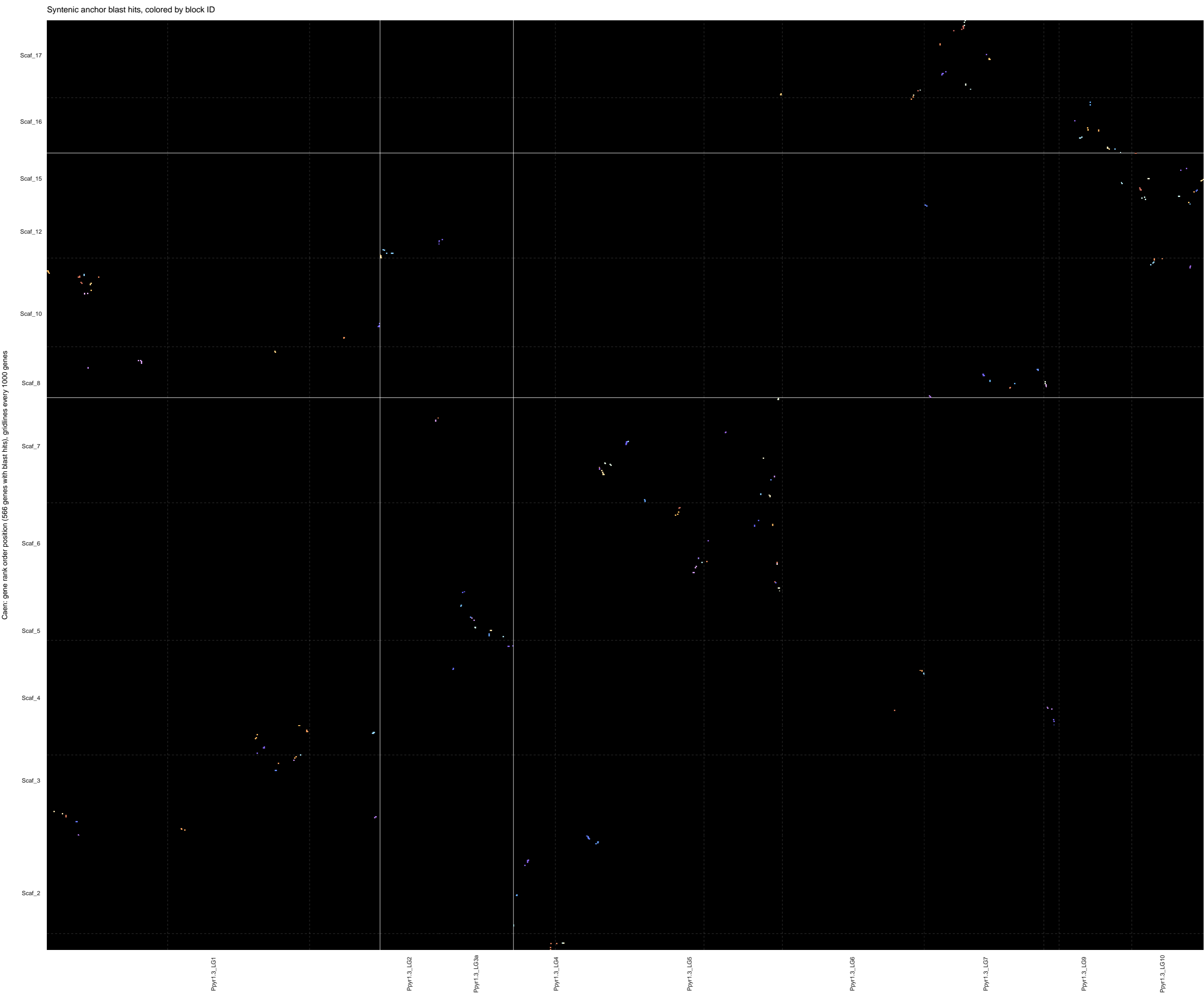

Supplement: S12 Fig — (PDF) [file pgen.1011477.s014.pdf]

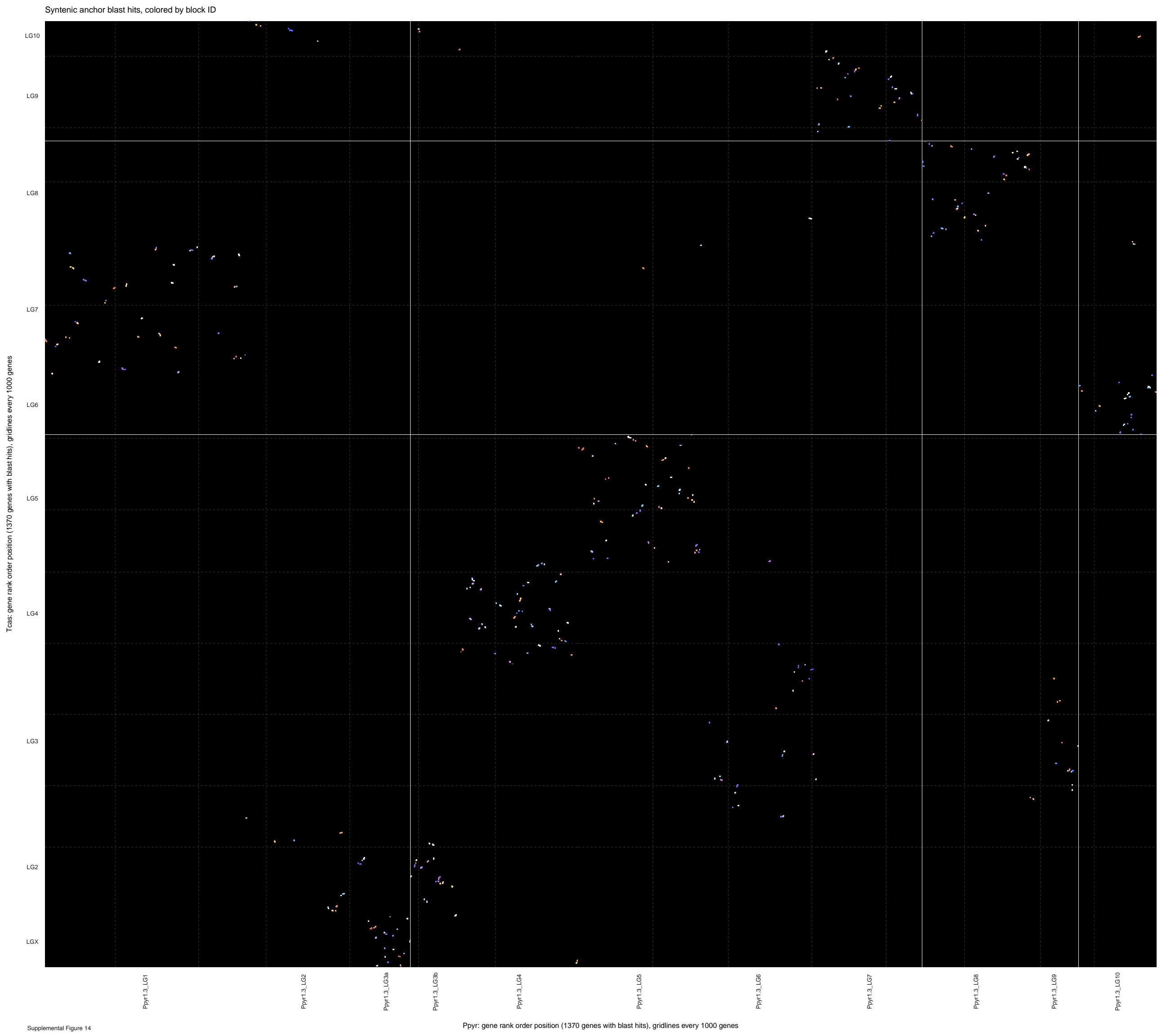

Supplement: S14 Fig — (PDF) [file pgen.1011477.s016.pdf]

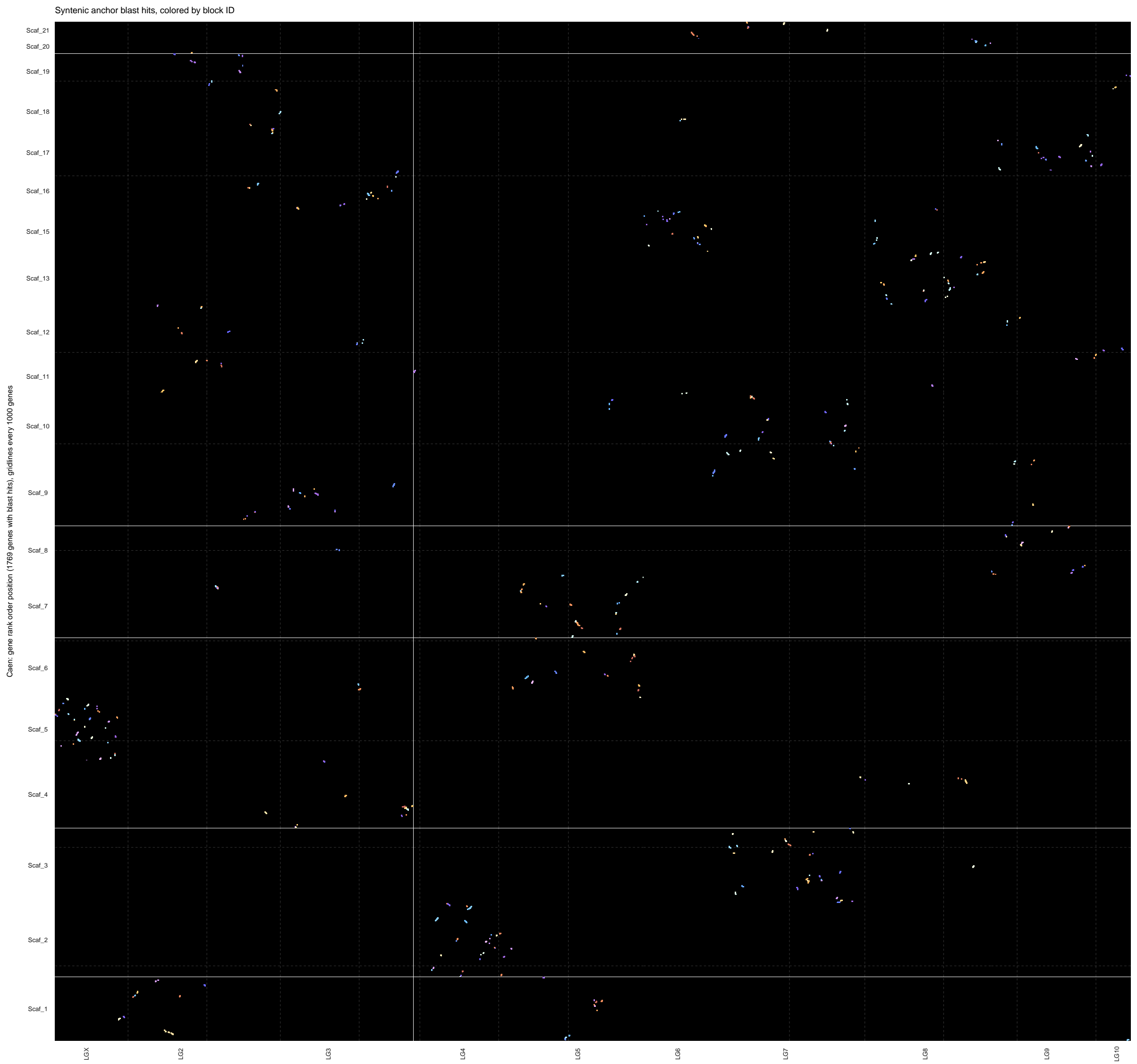

Supplement: S15 Fig — (PDF) [file pgen.1011477.s017.pdf]
