# Supplementary material for: Sex and neo-sex chromosome evolution in beetles
Source: PLoS Genet. 2024 Nov 25;20(11):e1011477. doi: 10.1371/journal.pgen.1011477 (PMC11753715; doi:10.1371/journal.pgen.1011477)
Supplement: S10 Fig — (PDF) [file pgen.1011477.s012.pdf]

Syntenic anchor blast hits, colored by block ID

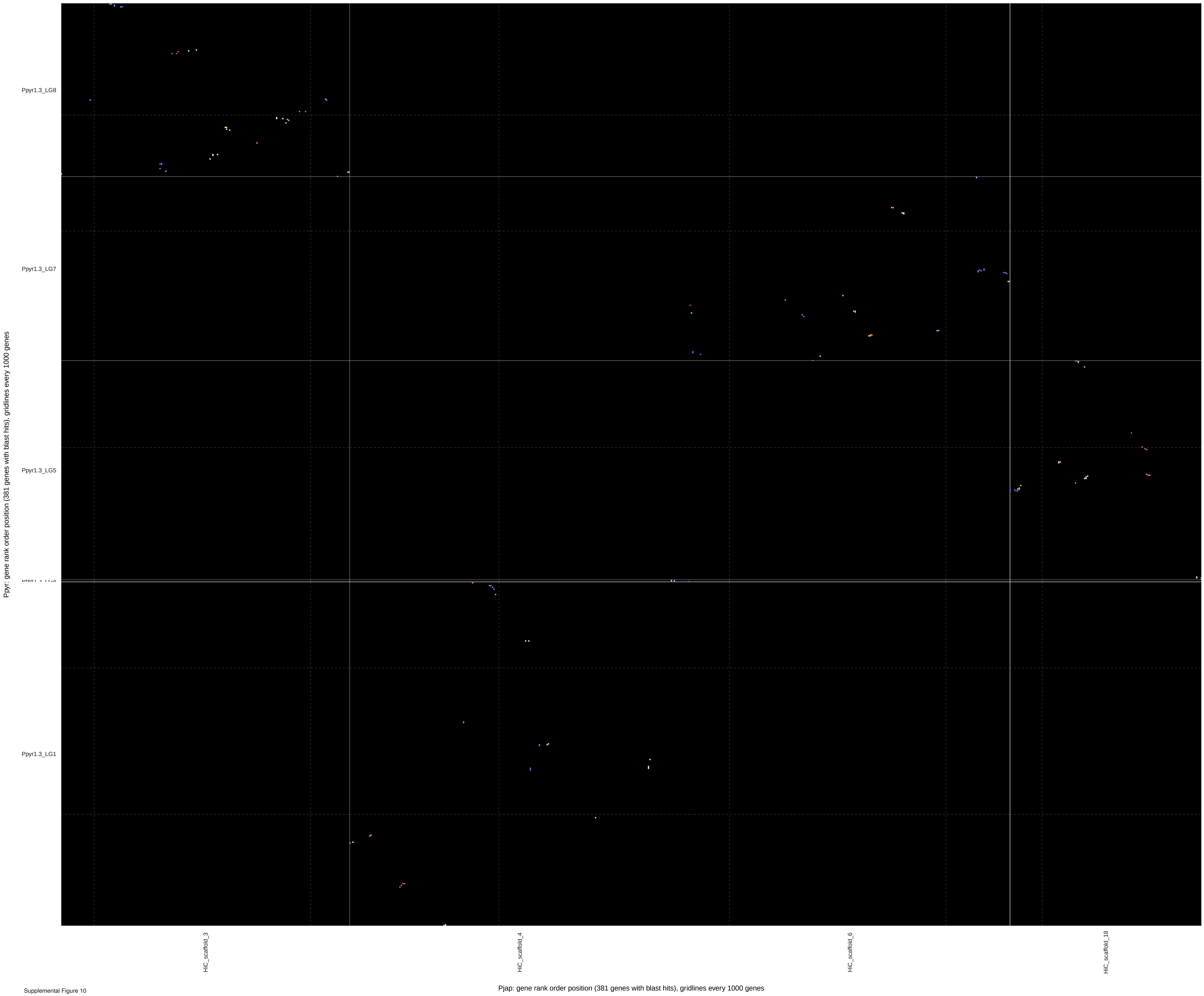

Supplemental Figure 10
