# Supplementary material for: Sex and neo-sex chromosome evolution in beetles
Source: PLoS Genet. 2024 Nov 25;20(11):e1011477. doi: 10.1371/journal.pgen.1011477 (PMC11753715; doi:10.1371/journal.pgen.1011477)
Supplement: S1 Note — (PDF) [file pgen.1011477.s018.pdf]

Supplementary Note 1. Y-linked gene analysis

While the genome assembly and annotation for the X and autosomes of *T. confusum* are of high quality, we are less confident in our assembly and annotation of the repeat-rich Y. Despite its relatively small size, there are 35 stitch points for the Y, i.e. its assembly is much more fragmented than for the X or autosomes (**Figure 2**). Male coverage for Y-linked scaffolds should be half that for autosomes when sequencing a male; however, most Y scaffolds show drastically increased coverage compared to autosomal or X-linked scaffolds (**Supplemental Figure 1**). This suggests that that many Y-linked scaffolds consist of collapsed repeats that are highly enriched on the Y chromosome, but which could not be correctly resolved by the genome assembler.

Also, despite our best effort to repeat-mask the genome before gene annotation, most of the Y genes identified contain (large segments) of repetitive DNA (**Appendix 1**).

Furthermore, many of the putative Y genes are not expressed in any of the tissues sampled (**Table A1**). Thus, many of these genes may not represent bone fide genes.

We used BLASTp to identify homologs for the 24 Y-linked putative proteins (>75% sequence similarity and >75% length cutoff; **Table A2**). Roughly half of the Y genes (11 out of 24) have a homolog on the X, consistent with the idea that these genes were ancestrally present on the chromosomes that formed the sex chromosomes in *T. confusum* (i.e., chromosome X and chromosome 2).

Table A1. Expression of Y-linked genes in different tissues (FPKM).

gene	male_head	female_head	Ovary_1	Ovary_2	Teste_1	Teste_2
ChrY-exonerate_est2genome-gene-0.0	0.1872	0.377838	0.335496	0.265472	0.093978	0.088126
ChrY-exonerate_est2genome-gene-1.2	2.501409	3.222251	13.49345	22.68034	1.69259	1.887435
ChrY-exonerate_protein2genome-gene-0.10	0	0	0	0	0	0
ChrY-exonerate_protein2genome-gene-0.11	0.327396	0	0	0	0	0
ChrY-exonerate_protein2genome-gene-0.12	0	0	0	0	0	0
ChrY-exonerate_protein2genome-gene-0.8	0.26288	0	0	0	0	0
ChrY-exonerate_protein2genome-gene-0.9	0	0	0	0	0	0
ChrY-exonerate_protein2genome-gene-1.10	0.030742	0	0.052805	0.072152	0.096197	0.17489
ChrY-exonerate_protein2genome-gene-1.11	0.204639	0	0	0	0	0
ChrY-exonerate_protein2genome-gene-1.12	0.505909	0.305408	0.469362	0.322078	0.183672	0.19857
ChrY-exonerate_protein2genome-gene-1.16	0.034292	0.062887	0	0	0.016806	0
ChrY-exonerate_protein2genome-gene-1.9	0	0	0	0	0	0.781843
ChrY-snap-gene-0.13	0	0.143287	0.098254	0.101048	0	0.021593
ChrY-snap-gene-0.15	0.105956	0.071681	0.19229	0.106773	0	0.160903
ChrY-snap-gene-0.16	0	0	0	0.091237	0	0
ChrY-snap-gene-0.17	0.07821	0.150629	0.053155	0	0	0
ChrY-snap-gene-1.17	0	0.395068	0	0	0.353594	0
ChrY-snap-gene-1.18	0.029352	0.086593	0	0.094075	0	0.035621
ChrY-snap-gene-1.20	0.512618	1.840964	3.08307	1.820343	0.106237	1.531095
ChrY-snap-gene-1.22	0.062391	0.119468	0.052753	0	0	0
ChrY-snap-gene-1.23	0.095769	0.212417	0	0.214238	0	0.22916
ChrY-snap-gene-1.25	0	0	0	0	0	0
ChrY-snap-gene-1.27	0.4256	1.640222	3.557706	4.008453	0.210574	1.473434
ChrY-snap-gene-1.28	0.275958	0.007799	1.454787	2.202458	1.069289	0.63969

Table A2. Blastp results for Y-linked genes

Chr Y query gene name	Blast result gene name	% identity	alignment ler	mismatches	gap opens	q. start	q. end	s. start	s. end	eval	bit score	Chrom
ChrY-exonerate_est2genome-gene-0.0	ChrY-exonerate_est2genome-gene-0.0	100	142	0	0	1	142	1	142	4.93E-102	287	self
ChrY-exonerate_est2genome-gene-0.0	ChrX_Chr2-exonerate_est2genome-gene-33.30	86.957	138	16	2	3	139	5	141	1.34E-79	231	X_Chr2
ChrY-exonerate_est2genome-gene-0.0	Chr4-exonerate_est2genome-gene-1.5	93.86	114	6	1	27	139	1	114	3.55E-71	209	4
ChrY-exonerate_est2genome-gene-0.0	Chr9_1-exonerate_est2genome-gene-0.0	96.19	105	3	1	1	104	94	198	4.51E-69	206	9_1
ChrY-snap-gene-0.17	ChrY-snap-gene-0.17	100	395	0	0	1	395	1	395	0	830	self
ChrY-snap-gene-0.17	ChrY-snap-gene-1.22	85.372	417	22	4	18	395	95	511	0	716	Y
ChrY-snap-gene-0.17	Chr8-exonerate_protein2genome-gene-11.41	84.539	401	23	4	34	395	1	401	0	676	8
ChrY-snap-gene-0.17	ChrX_Chr2-exonerate_protein2genome-gene-47.50	84.539	401	23	4	34	395	1	401	0	676	X_Chr2
ChrY-snap-gene-0.13	ChrY-snap-gene-0.13	100	236	0	0	1	236	1	236	2.88E-177	484	self
ChrY-snap-gene-0.13	Chr8-snap-gene-23.85	74.615	130	33	0	105	234	24	153	1.57E-68	206	8
ChrY-snap-gene-0.15	ChrY-snap-gene-0.15	100	163	0	0	1	163	1	163	1.21E-121	338	self
ChrY-exonerate_protein2genome-gene-0.8	ChrY-exonerate_protein2genome-gene-0.8	100	135	0	0	1	135	1	135	1.12E-99	280	
ChrY-exonerate_protein2genome-gene-0.8	ChrX_Chr2-exonerate_protein2genome-gene-49.32	92.784	97	7	0	13	109	1	97	8.88E-65	191	X_Chr2
ChrY-snap-gene-0.16	ChrY-snap-gene-0.16	100	91	0	0	1	91	1	91	2.81E-63	185	self
ChrY-snap-gene-0.16	Chr4-snap-gene-25.186	85	100	6	1	1	91	1	100	2.79E-55	168	4
ChrY-snap-gene-0.16	Chr4-snap-gene-3.141	85	100	6	1	1	91	1	100	3.12E-55	168	4
ChrY-snap-gene-0.16	Chr8-snap-gene-26.124	84	100	7	1	1	91	1	100	1.09E-54	167	8
ChrY-snap-gene-0.16	Chr7_2-snap-gene-14.408	82	100	9	1	1	91	1	100	2.85E-53	165	7_2
ChrY-snap-gene-0.16	ChrX_Chr2-snap-gene-13.176	83	100	7	2	1	91	1	99	5.47E-53	161	X_Chr2
ChrY-exonerate_protein2genome-gene-0.10	ChrY-exonerate_protein2genome-gene-0.10	100	84	0	0	1	84	1	84	4.07E-60	176	self
ChrY-exonerate_protein2genome-gene-0.10	ChrX_Chr2-exonerate_protein2genome-gene-1.62	89.286	84	9	0	1	84	1	84	3.52E-55	164	X_Chr2
ChrY-exonerate_protein2genome-gene-0.10	ChrX_Chr2-exonerate_protein2genome-gene-1.61	91.667	84	7	0	1	84	1	84	5.05E-55	163	X_Chr2
ChrY-exonerate_protein2genome-gene-0.10	Chr3-exonerate_protein2genome-gene-13.109	92.771	83	6	0	1	83	1	83	8.85E-55	163	3
ChrY-exonerate_protein2genome-gene-0.10	Chr3-exonerate_protein2genome-gene-13.110	90.361	83	7	1	1	83	1	82	7.10E-53	158	3
ChrY-exonerate_protein2genome-gene-0.10	ChrX_Chr2-exonerate_protein2genome-gene-33.73	87.952	83	10	0	1	83	1	83	2.06E-51	154	X_Chr2
ChrY-exonerate_protein2genome-gene-0.9	ChrY-exonerate_protein2genome-gene-0.12	100	71	0	0	1	71	1	71	1.43E-48	146	self
ChrY-exonerate_protein2genome-gene-0.9	ChrY-exonerate_protein2genome-gene-0.9	100	71	0	0	1	71	1	71	1.43E-48	146	Y
ChrY-exonerate_protein2genome-gene-0.9	ChrY-exonerate_protein2genome-gene-1.9	98.592	71	1	0	1	71	1	71	5.33E-48	145	Y
ChrY-exonerate_protein2genome-gene-0.9	Chr3-exonerate_protein2genome-gene-13.87	88.732	71	7	1	1	71	27	96	7.62E-41	127	3
ChrY-exonerate_protein2genome-gene-0.12	ChrY-exonerate_protein2genome-gene-0.12	100	71	0	0	1	71	1	71	1.43E-48	146	self
ChrY-exonerate_protein2genome-gene-0.12	ChrY-exonerate_protein2genome-gene-0.9	100	71	0	0	1	71	1	71	1.43E-48	146	Y
ChrY-exonerate_protein2genome-gene-0.12	ChrY-exonerate_protein2genome-gene-1.9	98.592	71	1	0	1	71	1	71	5.33E-48	145	Y
ChrY-exonerate_protein2genome-gene-0.12	Chr3-exonerate_protein2genome-gene-13.87	88.732	71	7	1	1	71	27	96	7.62E-41	127	3
ChrY-exonerate_protein2genome-gene-0.11	ChrY-exonerate_protein2genome-gene-0.11	100	27	0	0	1	27	1	27	4.49E-14	56.2	self
ChrY-snap-gene-1.18	ChrY-snap-gene-1.18	100	128	0	0	1	128	1	128	1.78E-93	264	self
ChrY-snap-gene-1.18	ChrY-snap-gene-1.23	99.13	115	0	1	1	115	1	114	1.39E-81	234	Y
ChrY-snap-gene-1.18	ChrY-snap-gene-1.25	98.261	115	1	1	1	115	1	114	6.33E-81	232	Y

ChrY-snap-gene-1.18	Chr8-snap-gene-15.132	86.719	128	15	2	1	128	18	143	3.10E-75	218	8
ChrY-snap-gene-1.18	ChrX_Chr2-snap-gene-48.84	92.174	115	8	1	1	115	1	114	5.26E-73	212	X_Chr2
ChrY-exonerate_est2genome-gene-1.2	ChrY-exonerate_est2genome-gene-1.2	100	278	0	0	1	278	1	278	0	575	self
ChrY-snap-gene-1.23	ChrY-snap-gene-1.23	100	126	0	0	1	126	1	126	9.39E-93	262	self
ChrY-snap-gene-1.23	ChrY-snap-gene-1.25	98.413	126	2	0	1	126	1	126	2.05E-91	259	Y
ChrY-snap-gene-1.23	ChrX_Chr2-snap-gene-48.84	92.8	125	9	0	1	125	1	125	1.04E-82	236	X_Chr2
ChrY-snap-gene-1.23	Chr8-snap-gene-15.132	93.443	122	8	0	1	122	18	139	2.72E-82	236	8
ChrY-snap-gene-1.23	ChrY-snap-gene-1.18	99.13	115	0	1	1	114	1	115	1.37E-81	234	Y
ChrY-snap-gene-1.25	ChrY-snap-gene-1.25	100	126	0	0	1	126	1	126	2.31E-92	261	self
ChrY-snap-gene-1.25	ChrY-snap-gene-1.23	98.413	126	2	0	1	126	1	126	2.05E-91	259	Y
ChrY-snap-gene-1.25	ChrX_Chr2-snap-gene-48.84	91.2	125	11	0	1	125	1	125	2.03E-81	233	X_Chr2
ChrY-snap-gene-1.25	Chr8-snap-gene-15.132	91.803	122	10	0	1	122	18	139	4.55E-81	233	8
ChrY-snap-gene-1.25	ChrY-snap-gene-1.18	98.261	115	1	1	1	114	1	115	6.23E-81	232	Y
ChrY-snap-gene-1.27	ChrY-snap-gene-1.27	100	416	0	0	1	416	1	416	0	865	self
ChrY-snap-gene-1.27	ChrX_Chr2-snap-gene-2.171	92.78	277	1	1	159	416	225	501	2.36E-177	503	X_Chr2
ChrY-snap-gene-1.27	ChrX_Chr2-snap-gene-2.171	100	154	0	0	1	154	1	154	7.76E-106	320	X_Chr2
ChrY-snap-gene-1.22	ChrY-snap-gene-1.22	100	511	0	0	1	511	1	511	0	1068	self
ChrY-snap-gene-1.22	Chr8-exonerate_protein2genome-gene-11.41	99.002	401	4	0	111	511	1	401	0	830	8
ChrY-snap-gene-1.22	ChrX_Chr2-exonerate_protein2genome-gene-47.50	99.002	401	4	0	111	511	1	401	0	830	X_Chr2
ChrY-snap-gene-1.22	ChrY-snap-gene-0.17	85.372	417	22	4	95	511	18	395	0	716	Y
ChrY-exonerate_protein2genome-gene-1.16	ChrY-exonerate_protein2genome-gene-1.16	100	269	0	0	1	269	1	269	0	548	self
ChrY-exonerate_protein2genome-gene-1.16	Chr4-exonerate_est2genome-gene-1.0	52.918	257	121	0	11	267	253	509	1.56E-93	283	4
ChrY-exonerate_protein2genome-gene-1.16	Chr6-exonerate_est2genome-gene-23.14	52.529	257	122	0	11	267	253	509	1.36E-92	281	6
ChrY-snap-gene-1.20	ChrY-snap-gene-1.20	100	223	0	0	1	223	1	223	1.12E-166	457	self
ChrY-exonerate_protein2genome-gene-1.10	ChrY-exonerate_protein2genome-gene-1.10	100	195	0	0	3	197	3	197	8.48E-143	394	self
ChrY-exonerate_protein2genome-gene-1.10	ChrY-exonerate_protein2genome-gene-1.16	100	119	0	0	75	193	1	119	1.83E-81	242	Y
ChrY-snap-gene-1.28	ChrY-snap-gene-1.28	100	193	0	0	1	193	1	193	4.16E-143	395	self
ChrY-snap-gene-1.28	ChrX_Chr2-snap-gene-2.169	78.882	161	3	3	35	187	1	138	1.50E-82	239	X_Chr2
ChrY-exonerate_protein2genome-gene-1.11	ChrY-exonerate_protein2genome-gene-1.11	100	98	0	0	1	98	1	98	2.44E-70	203	self
ChrY-exonerate_protein2genome-gene-1.9	ChrY-exonerate_protein2genome-gene-1.9	100	72	0	0	1	72	1	72	1.09E-49	149	self
ChrY-exonerate_protein2genome-gene-1.9	ChrY-exonerate_protein2genome-gene-0.12	98.592	71	1	0	1	71	1	71	5.40E-48	145	Y
ChrY-exonerate_protein2genome-gene-1.9	ChrY-exonerate_protein2genome-gene-0.9	98.592	71	1	0	1	71	1	71	5.40E-48	145	Y
ChrY-exonerate_protein2genome-gene-1.9	Chr3-exonerate_protein2genome-gene-13.87	90.278	72	6	1	1	72	27	97	1.82E-42	132	3
ChrY-snap-gene-1.17	ChrY-snap-gene-1.17	100	64	0	0	1	64	1	64	1.65E-42	130	self
ChrY-snap-gene-1.17	Chr6-exonerate_protein2genome-gene-18.52	94.643	56	3	0	1	56	1	56	2.61E-35	113	6
ChrY-exonerate_protein2genome-gene-1.12	ChrY-exonerate_protein2genome-gene-1.12	100	47	0	0	1	47	1	47	7.26E-29	95.1	self

Appendix 1 – Repeat masking of putative Y-linked genes using Repbase (https://www.girinst.org/)

maker-ChrY-exonerate_est2genome-gene-0.0 (SVG Plot; Alignments; Masked)

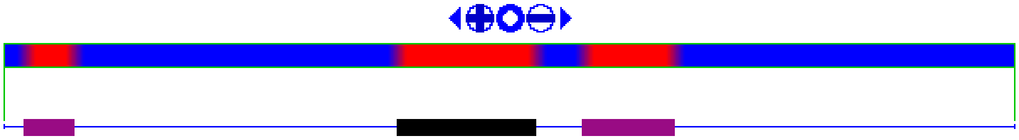

Name	From	To	Name	From	To	Class	Dir	Sim	Pos/Mm:Ts	Score
maker-ChrY-exonerate_est2genome-gene-0.0	20	64	HARB2_ZM	3324	3370	DNA/Harbinger	d	0.8261	3.5000	240
maker-ChrY-exonerate_est2genome-gene-0.0	352	476	Caulimovirus-1_MT	167	307	IntegratedVirus/Caulimovirus	d	0.7176	1.4444	207
maker-ChrY-exonerate_est2genome-gene-0.0	518	599	Mariner-40_CorFlu	476	551	DNA/Mariner	d	0.7436	1.8889	223

maker-ChrY-exonerate_est2genome-gene-1.2 (SVG Plot; Alignments; Masked)

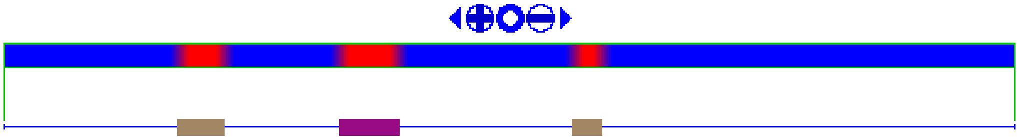

Name	From	To	Name	From	To	Class	Dir	Sim	Pos/Mm:Ts	Score
maker-ChrY-exonerate_est2genome-gene-1.2	158	199	Gypsy-9_GeSe-I	60	101	LTR/Gypsy	d	0.7619	1.1111	215
maker-ChrY-exonerate_est2genome-gene-1.2	303	356	Polinton-2_PH	8037	8089	DNA/Polinton	c	0.7778	1.4286	227
maker-ChrY-exonerate_est2genome-gene-1.2	513	539	BEL-42_CGi-I	965	991	LTR/BEL	c	0.9630	1.0000	220

maker-ChrY-exonerate_protein2genome-gene-0.12 (SVG Plot; Alignments; Masked)

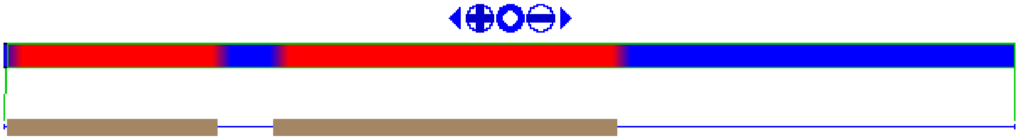

Name	From	To	Name	From	To	Class	Dir	Sim	Pos/Mm:Ts	Score
maker-ChrY-exonerate_protein2genome-gene-0.12	2	46	DIRS-18_PhPy	4095	4139	LTR/DIRS	c	0.7778	2.0000	230
maker-ChrY-exonerate_protein2genome-gene-0.12	58	130	DIRS-8_SiOr	4302	4372	LTR/DIRS	c	0.7671	1.7500	273

maker-ChrY-exonerate_protein2genome-gene-0.8 (SVG Plot; Alignments; Masked)

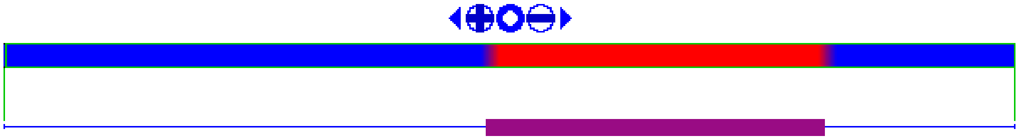

Name	From	To	Name	From	To	Class	Dir	Sim	Pos/Mm:Ts	Score
maker-ChrY-exonerate_protein2genome-gene-0.8	196	332	Merlin-1_DF	932	1065	DNA/Merlin	d	0.6767	1.8182	319

maker-ChrY-exonerate_protein2genome-gene-0.9 (SVG Plot; Alignments; Masked)

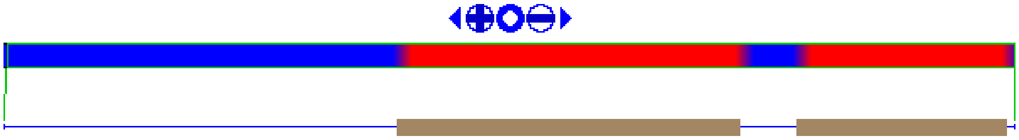

Name	From	To	Name	From	To	Class	Dir	Sim	Pos/Mm:Ts	Score
maker-ChrY-exonerate_protein2genome-gene-0.9	84	156	DIRS-8_SiOr	4302	4372	LTR/DIRS	d	0.7671	1.7500	270
maker-ChrY-exonerate_protein2genome-gene-0.9	168	212	DIRS-18_PhPy	4095	4139	LTR/DIRS	d	0.7778	2.0000	230

maker-ChrY-exonerate_protein2genome-gene-1.10 (SVG Plot; Alignments; Masked)

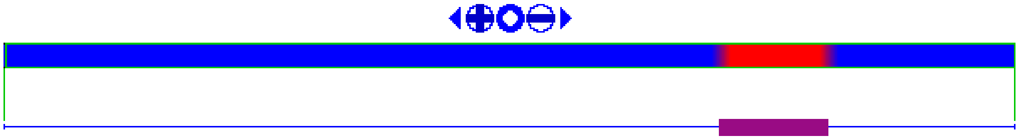

Name	From	To	Name	From	To	Class	Dir	Sim	Pos/Mm:Ts	Score
maker-ChrY-exonerate_protein2genome-gene-1.10	421	485	Sola3-2_NV	1053	1115	DNA/Sola/Sola3	d	0.7656	1.4444	259

maker-ChrY-exonerate_protein2genome-gene-1.11 (SVG Plot; Alignments; Masked)

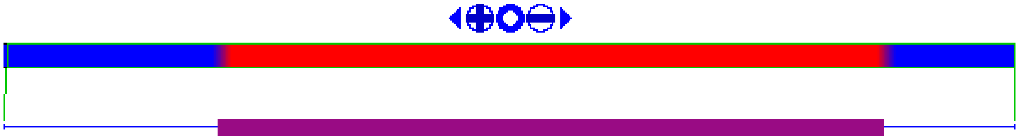

Name	From	To	Name	From	To	Class	Dir	Sim	Pos/Mm:Ts	Score
maker-ChrY-exonerate_protein2genome-gene-1.11	64	259	Sailor-Mo-Halrub	952	1141	DNA/Mariner	c	0.6963	2.0800	423

maker-ChrY-exonerate_protein2genome-gene-1.16 (SVG Plot; Alignments; Masked)

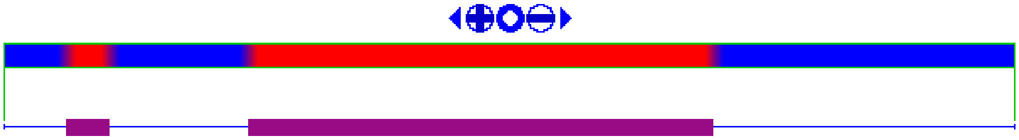

Name	From	To	Name	From	To	Class	Dir	Sim	Pos/Mm:Ts	Score
maker-ChrY-exonerate_protein2genome-gene-1.16	51	86	VANDAL16	9622	9657	DNA/MuDR	d	0.8919	2.0000	226
maker-ChrY-exonerate_protein2genome-gene-1.16	197	569	ISL2EU-15_CorFlu	1488	1860	DNA/ISL2EU	c	0.6595	1.8103	481

maker-ChrY-exonerate_protein2genome-gene-1.9 (SVG Plot; Alignments; Masked)

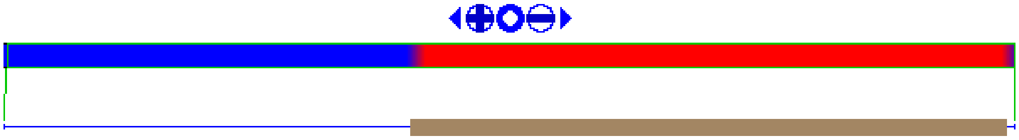

Name	From	To	Name	From	To	Class	Dir	Sim	Pos/Mm:Ts	Score
maker-ChrY-exonerate_protein2genome-gene-1.9	88	215	DIRS-18_PhPy	4018	4142	LTR/DIRS	d	0.7295	1.3158	349

maker-ChrY-snap-gene-0.13 (SVG Plot; Alignments; Masked)

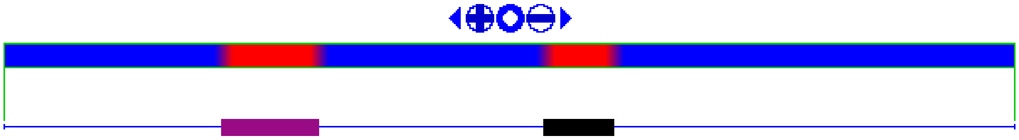

Name	From	To	Name	From	To	Class	Dir	Sim	Pos/Mm:Ts	Score
maker-ChrY-snap-gene-0.13	155	222	Helitron-6_ALy	7296	7366	DNA/Helitron	d	0.8000	1.3333	221
maker-ChrY-snap-gene-0.13	381	429	Caulimovirus-1_ADu	11290	11337	IntegratedVirus/Caulimovirus	d	0.7959	1.8000	213

maker-ChrY-snap-gene-0.15 (SVG Plot; Alignments; Masked)

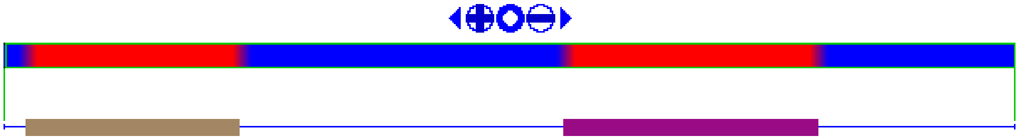

Name	From	To	Name	From	To	Class	Dir	Sim	Pos/Mm:Ts	Score
maker-ChrY-snap-gene-0.15	12	115	Gypsy-17_DBi-I	3035	3129	LTR/Gypsy	c	0.7245	1.9167	257
maker-ChrY-snap-gene-0.15	272	394	SENKUSHA1	1174	1293	DNA/Harbinger	c	0.6833	1.8421	361

maker-ChrY-snap-gene-0.17 (SVG Plot; Alignments; Masked)

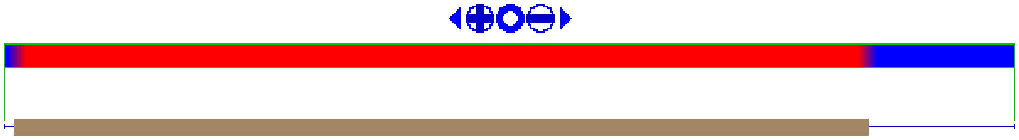

Name	From	To	Name	From	To	Class	Dir	Sim	Pos/Mm:Ts	Score
maker-ChrY-snap-gene-0.17	14	1013	Gypsy-91_RhyDom-I	3862	4856	LTR/Gypsy	c	0.6421	1.8485	1193

maker-ChrY-snap-gene-1.18 (SVG Plot; Alignments; Masked)

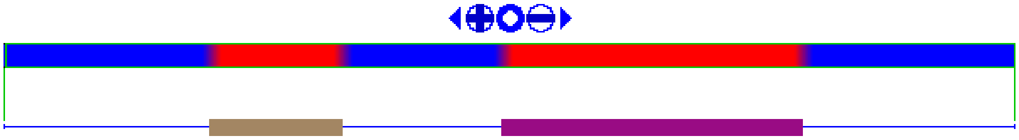

Name	From	To	Name	From	To	Class	Dir	Sim	Pos/Mm:Ts	Score
maker-ChrY-snap-gene-1.18	89	145	GYVIT1_I	103	159	LTR/Gypsy	c	0.8070	2.0000	235
maker-ChrY-snap-gene-1.18	213	341	ISL2EU-29_CGi	3177	3314	DNA/ISL2EU	d	0.7680	1.9091	293

maker-ChrY-snap-gene-1.20 (SVG Plot; Alignments; Masked)

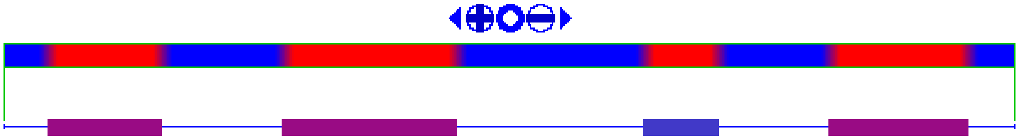

Name	From	To	Name	From	To	Class	Dir	Sim	Pos/Mm:Ts	Score
maker-ChrY-snap-gene-1.20	30	105	Transib-4_DBi	1288	1365	DNA/Transib	d	0.7143	1.8182	237
maker-ChrY-snap-gene-1.20	186	300	hATm-17_HM	700	808	DNA/hAT	d	0.7143	2.0000	248
maker-ChrY-snap-gene-1.20	424	473	CasCan-3.129	2119	2167	NonLTR/L1	d	0.8542	1.6667	258
maker-ChrY-snap-gene-1.20	547	638	hAT-1_Cas	3349	3438	DNA/hAT	c	0.7753	2.3333	206

maker-ChrY-snap-gene-1.22 (SVG Plot; Alignments; Masked)

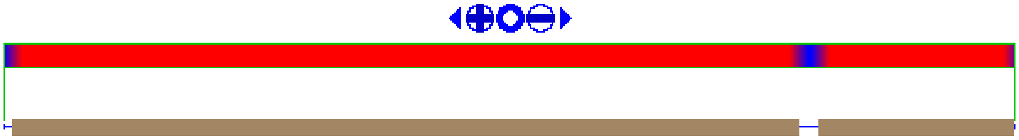

Name	From	To	Name	From	To	Class	Dir	Sim	Pos/Mm:Ts	Score
maker-ChrY-snap-gene-1.22	14	1223	Gypsy-19_SiOr-I	3762	4972	LTR/Gypsy	c	0.6576	2.0296	1171
maker-ChrY-snap-gene-1.22	1256	1552	Gypsy-8_RhyDom-I	2421	2717	LTR/Gypsy	c	0.7085	1.9000	840

maker-ChrY-snap-gene-1.23 (SVG Plot; Alignments; Masked)

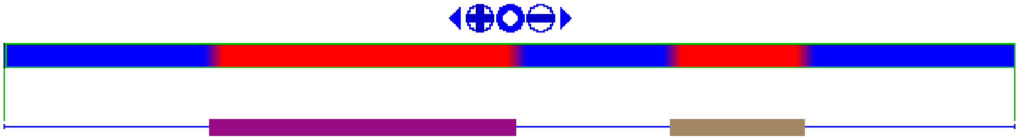

Name	From	To	Name	From	To	Class	Dir	Sim	Pos/Mm:Ts	Score
maker-ChrY-snap-gene-1.23	88	216	ISL2EU-29_CGi	3177	3314	DNA/ISL2EU	c	0.7680	1.9091	293
maker-ChrY-snap-gene-1.23	281	337	GYVIT1_I	103	159	LTR/Gypsy	d	0.8070	2.0000	235

maker-ChrY-snap-gene-1.25 (SVG Plot; Alignments; Masked)

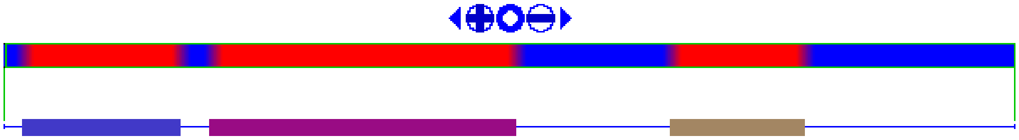

Name	From	To	Name	From	To	Class	Dir	Sim	Pos/Mm:Ts	Score
maker-ChrY-snap-gene-1.25	9	75	I-2_DK	2479	2539	NonLTR/I	d	0.7903	1.4286	217
maker-ChrY-snap-gene-1.25	88	216	ISL2EU-29_CGi	3177	3314	DNA/ISL2EU	c	0.7680	1.9091	293
maker-ChrY-snap-gene-1.25	281	337	GYVIT1_I	103	159	LTR/Gypsy	d	0.8070	2.0000	235

maker-ChrY-snap-gene-1.27 (SVG Plot; Alignments; Masked)

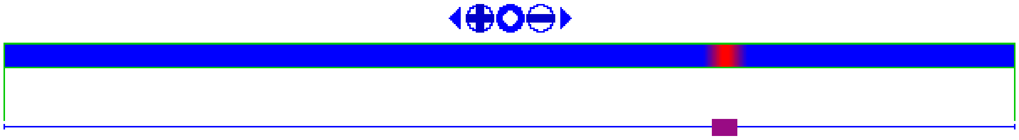

Name	From	To	Name	From	To	Class	Dir	Sim	Pos/Mm:Ts	Score
maker-ChrY-snap-gene-1.27	877	907	hAT-N2_AmAm	1330	1359	DNA/hAT	d	0.9355	1.0000	221

maker-ChrY-snap-gene-1.28 (SVG Plot; Alignments; Masked)

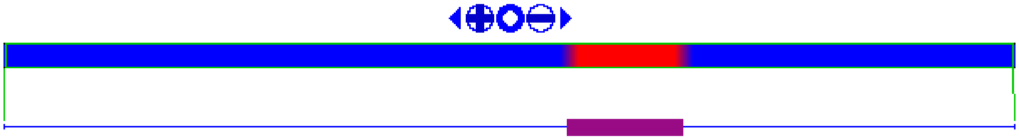

Name	From	To	Name	From	To	Class	Dir	Sim	Pos/Mm:Ts	Score
maker-ChrY-snap-gene-1.28	325	391	CryptonV-4_CorFlu	6115	6177	DNA/Crypton/CryptonV	d	0.7538	2.3333	230

Masked Sequence

```
>maker-ChrY-snap-gene-0.13
ATGTCAAAAATTGTTATCAAAATGTACTTTTGGGTTTATGTTTTGTGTACTTTTGTAATGGCGGTTTTTG
CGTGGTTATCTGGTAAAGATTTTCCATCAAATCATTGGATACCGTTTGAATACCACAAACCGTTTCTGTT
TCAAATTATTTATTXXXXXXXXXXXXXXXXXXXXXXXXXXXXXXXXXXXXXXXXXXXXXXXXXXXXXXXX
XXXXXXXXXXXXTCTTTGGGACAGATTACAGCGCAGTGTGATATTCTTGCAAATACTCTACGAAATATAC
```

ATGATCTAAAGAAACTAAATGTTGCACATAAAGTCACAAAATATAAAAATAAAGATGAGGTTATGAATGA
TGTACTAGCAGAGTGTGTACAACATTATAAXXXXXXXXXXXXXXXXXXXXXXXXXXXXXXXXXXXXXXXX
XXXXXXXXXGTGACTTTTCAGTTTATACCGTCAGTTCTTATGATTTGTTTTGCAATGTTCAAAGTATCTT
CGAGTGAAGAACTATTTTACGCTGCTTTTGAATCAGAGTGGTATAATGCATCACAAAAATTTAAGAAAAA
TTTGTTAACAATGATGATTATGGTACAAAAACCGATAGTTTTTTATAGTTGGGATATTTTTGCAGTAAAT
TATGAAGCTTTTAAATCGATTATGCAAAAATCGTGGAGCATCTGTATTGCTCTTAAAAGCACACAAGATT
TGAACCAGTAG
>maker-ChrY-exonerate_protein2genome-gene-0.10
TTAATTCTTATTCGCTTTTTCACAAGCATCAATCCAGTCGTTGTACACATCAATGGGGTCCGAAAGATAG
TTAATAGTCGTTTGGAAATCTTCTCCACAAACTCCACAGGTTATTTTCGCTGCATTTCTTCCCTTATCTA
TTAAAACCGTGCAGGACTTTTTGTGGTTACAAAAAGGACAGTTAAATTCCTTATCGAGGGGTTGCAAAGC
TCTCCTTCTTGGTGGTGGCTTTCTTTTCAATTTGCTGCGTCCCAT
>maker-ChrY-exonerate_est2genome-gene-0.0
GACGGAAATGTGAGTATTTXXXXXXXXXXXXXXXXXXXXXXXXXXXXXXXXXXXXXXXXXXXXXXACTTTT
GAAAAAACTTGTTGTTTTATGTAGGTGTTATAAATATATAACTAATCGGAAATTGTATTTTTAAGCATGA
GTGAAAGCGGTACCATTAAAACGAAAAAGAGCTGCAAATTTTACAGAACTGGAAACAAGGATCTTGGTGG
ATGTTGTATTACGACATATTGATATAATTGAAAATAAGCGCAGCGATAGCATAACATGGAAGCAGAAGTC
AGATACTTGGGAAATAATCGCCAACCAGTTTAACAGTTTAACCGGATCACGAAACAGACCAGCAAAAGAA
TXXXXXXXXXXXXXXXXXXXXXXXXXXXXXXXXXXXXXXXXXXXXXXXXXXXXXXXXXXXXXXXXXXXXX
XXXXXXXXXXXXXXXXXXXXXXXXXXXXXXXXXXXXXXXXXXXXXXXXXXXXXXXXGATTTTAAGTCACA
TTATGTTAACAGTTGAAGGTTTGATTGXXXXXXXXXXXXXXXXXXXXXXXXXXXXXXXXXXXXXXXXXXX
XXXXXXXXXXXXXXXXXXXXXXXXXXXXXXXXXXXXXXXTCAAGTTATAAATTCCTTATTTACTTCAGCT
GCCTTTAATGTGCCCACATCAGTAAGCAGTGTAACGACTACATCTGGCTCTCAATCTGCGACAACTGTGT
TAAATGTTCAGAAAACTGTCTATAGTTTATATACACCGGTAACAGAGTCAGACACTACAGTCACCCTTCT
ATCACCTTGTTTGCCCAAGACTAATAATGACACAACAATTAACCAAACGAATTTTTTGGCAGTTTTCTCA
TAAGACTACTAAAAGTATGGCAATTAATATGCAAGATACCAACCCGATCGAAGAAACTAAGGT
>maker-ChrY-snap-gene-0.15
AATATGATTGCXXXXXXXXXXXXXXXXXXXXXXXXXXXXXXXXXXXXXXXXXXXXXXXXXXXXXXXXXXX
XXXXXXXXXXXXXXXXXXXXXXXXXXXXXXXXXXXXXXXXXXXXXTTATAATGAGACTGGGCCCTCGTTA
AGTGTCCACGATCTTTAAAAGGAGTCATTAAGTTATTCAATAGTGGGTATCCACTGTCTCCCAGCAAGTA
GAAATTGTCACATTTTTGGTGAATACTCTCCCATAAAGGAGAGCTTCGAAAAAATTGACTAXXXXXXXXX
XXXXXXXXXXXXXXXXXXXXXXXXXXXXXXXXXXXXXXXXXXXXXXXXXXXXXXXXXXXXXXXXXXXXXX
XXXXXXXXXXXXXXXXXXXXXXXXXXXXXXXXXXXXXXXXXXXXGGTTTATCAATCTTGACGTGCGAACC
GTCTATTGCTCCGATAACCCCAAGAAACCCATTGTGACGAAAATGTCTTTCTATGTCATTTTTTTTCAT
>maker-ChrY-snap-gene-0.16
TCATAAGAACAGCTGTTTTTCTTCCATGACGTCTTTGATTATGTTGTAAAACTCTCGGCCTCGAATGAAT
GAATTATCCTGGATATCATCCTTGACTGCTGCAGCTTCATTAGAATTAATATTTTTGTCACAACAAATTA
CATCATCGTCGGAAAAAAAAGATAACTTAAGGCGATCTACACATCCAGGATGGAATATGCGACTAATTTT
TACACATTTGGGCCCTCTTAGGGCCTTATTACTACAATATAAACACTTCAATTCAGAGCTCGACAT
>maker-ChrY-exonerate_protein2genome-gene-0.11
TTAAAAAGAAATTCGGCCAAATAGCCCGCAAAGTGCCGAGATTTTCTTCCAAAACGAGGCACTGAACCAC
GGACGTCCCGCCAT
>maker-ChrY-exonerate_protein2genome-gene-0.8
GTTTTCGGTGGTTTTGACCGAACCACCAAAGAAATCTTTTTGGTACCCGTACCTCGTCGTGATGCGGAGA
CACTTCAAGATCTAATCCGTAGATGGATTAGACCTGGAAGTGTAATCGTATCCGATTGTTGGAAGGGATA
TGATAGATTAACTCAAATTGGATTCGAGCATTACCGCATTAACCATTCCTTTCAAXXXXXXXXXXXXXXX
XXXXXXXXXXXXXXXXXXXXXXXXXXXXXXXXXXXXXXXXXXXXXXXXXXXXXXXXXXXXXXXXXXXXXX
XXXXXXXXXXXXXXXXXXXXXXXXXXXXXXXXXXXXXXXXXXXXXXXXXXXXGAAGTTTAATCAACTAGA
AGAAAGGCTCCATCACATATCTCTTGCAGCTGCCGCAATGTATCTTCCACCTCCGTAA
>maker-ChrY-snap-gene-0.17
GTCTTTAACAATTXXXXXXXXXXXXXXXXXXXXXXXXXXXXXXXXXXXXXXXXXXXXXXXXXXXXXXXXX
XXXXXXXXXXXXXXXXXXXXXXXXXXXXXXXXXXXXXXXXXXXXXXXXXXXXXXXXXXXXXXXXXXXXXX
XXXXXXXXXXXXXXXXXXXXXXXXXXXXXXXXXXXXXXXXXXXXXXXXXXXXXXXXXXXXXXXXXXXXXX
XXXXXXXXXXXXXXXXXXXXXXXXXXXXXXXXXXXXXXXXXXXXXXXXXXXXXXXXXXXXXXXXXXXXXX
XXXXXXXXXXXXXXXXXXXXXXXXXXXXXXXXXXXXXXXXXXXXXXXXXXXXXXXXXXXXXXXXXXXXXX
XXXXXXXXXXXXXXXXXXXXXXXXXXXXXXXXXXXXXXXXXXXXXXXXXXXXXXXXXXXXXXXXXXXXXX
XXXXXXXXXXXXXXXXXXXXXXXXXXXXXXXXXXXXXXXXXXXXXXXXXXXXXXXXXXXXXXXXXXXXXX
XXXXXXXXXXXXXXXXXXXXXXXXXXXXXXXXXXXXXXXXXXXXXXXXXXXXXXXXXXXXXXXXXXXXXX
XXXXXXXXXXXXXXXXXXXXXXXXXXXXXXXXXXXXXXXXXXXXXXXXXXXXXXXXXXXXXXXXXXXXXX
XXXXXXXXXXXXXXXXXXXXXXXXXXXXXXXXXXXXXXXXXXXXXXXXXXXXXXXXXXXXXXXXXXXXXX
XXXXXXXXXXXXXXXXXXXXXXXXXXXXXXXXXXXXXXXXXXXXXXXXXXXXXXXXXXXXXXXXXXXXXX
XXXXXXXXXXXXXXXXXXXXXXXXXXXXXXXXXXXXXXXXXXXXXXXXXXXXXXXXXXXXXXXXXXXXXX
XXXXXXXXXXXXXXXXXXXXXXXXXXXXXXXXXXXXXXXXXXXXXXXXXXXXXXXXXXXXXXXXXXXXXX
XXXXXXXXXXXXXXXXXXXXXXXXXXXXXXXXXXXXXXXXXXXXXXXXXXXXXXXXXXXXXXXXXXXXXX
XXXXXXXXXXXXXXXXXXXXXXXXXXXXXXXXXACTTCAGGAGTATATTATGTTCAACGCGGTAAGTTGG
GTACTTTTCTGGATTTGATGTTACACTTTTAATCATTTCGGAGTACCACCGATCAGAAGTGTCGGACCAA
TTGATATCTAAATTACTCTCAGATTGAACAGAGTCAGTTAATACAGGAACAGTACGAGAGAGCAT
>maker-ChrY-exonerate_protein2genome-gene-0.12
GXXXXXXXXXXXXXXXXXXXXXXXXXXXXXXXXXXXXXXXXXXXXXTCACCAAAGGTXXXXXXXXXXXXX
XXXXXXXXXXXXXXXXXXXXXXXXXXXXXXXXXXXXXXXXXXXXXXXXXXXXXXXXXXXXTTTTGAAAAT
TTGCTTTAAGCGGTGCAGTTCTTCGAAGATAGGACTCCAACGTTGTCGCTACACAAATCTTAGGACTTTT
CAT
>maker-ChrY-exonerate_protein2genome-gene-0.9
ATGAAAAGTCCTAAGATTTGTGTAGCGACAACGTTGGAGTCCTATCTTCGAAGAACTGCACCGCTTAAAG
CAAATTTTCAAAAXXXXXXXXXXXXXXXXXXXXXXXXXXXXXXXXXXXXXXXXXXXXXXXXXXXXXXXXX
XXXXXXXXXXXXXXXXACCTTTGGTGAXXXXXXXXXXXXXXXXXXXXXXXXXXXXXXXXXXXXXXXXXXX
XXC

```
>maker-ChrY-exonerate_protein2genome-gene-1.11
CTACTGTTTTATTACTTTTTTACGTAAAGCACTAATAGATGGAGGAGTCTCAAAGCAAACTGAXXXXXXX
XXXXXXXXXXXXXXXXXXXXXXXXXXXXXXXXXXXXXXXXXXXXXXXXXXXXXXXXXXXXXXXXXXXXXX
XXXXXXXXXXXXXXXXXXXXXXXXXXXXXXXXXXXXXXXXXXXXXXXXXXXXXXXXXXXXXXXXXXXXXX
XXXXXXXXXXXXXXXXXXXXXXXXXXXXXXXXXXXXXXXXXXXXXXXXXGTATATCTTCTACCTTCACTC
TTGAAACTTGATAAAAT
>maker-ChrY-exonerate_protein2genome-gene-1.12
CTAATCCATGCGTGGCCAAATCATCACGTTTTCCTGCAGAGCCATTGATTGAGGCCCGACCCAAAGTTGT
GGAGGTTGAACTGTCATAGACAAGGCCTGGTTTATTTGCCATTTCATCAATAAGTAAGCAGGCATGCTTC
TCAT
>maker-ChrY-snap-gene-1.22
GTCTTTAACAATTXXXXXXXXXXXXXXXXXXXXXXXXXXXXXXXXXXXXXXXXXXXXXXXXXXXXXXXXX
XXXXXXXXXXXXXXXXXXXXXXXXXXXXXXXXXXXXXXXXXXXXXXXXXXXXXXXXXXXXXXXXXXXXXX
XXXXXXXXXXXXXXXXXXXXXXXXXXXXXXXXXXXXXXXXXXXXXXXXXXXXXXXXXXXXXXXXXXXXXX
XXXXXXXXXXXXXXXXXXXXXXXXXXXXXXXXXXXXXXXXXXXXXXXXXXXXXXXXXXXXXXXXXXXXXX
XXXXXXXXXXXXXXXXXXXXXXXXXXXXXXXXXXXXXXXXXXXXXXXXXXXXXXXXXXXXXXXXXXXXXX
XXXXXXXXXXXXXXXXXXXXXXXXXXXXXXXXXXXXXXXXXXXXXXXXXXXXXXXXXXXXXXXXXXXXXX
XXXXXXXXXXXXXXXXXXXXXXXXXXXXXXXXXXXXXXXXXXXXXXXXXXXXXXXXXXXXXXXXXXXXXX
XXXXXXXXXXXXXXXXXXXXXXXXXXXXXXXXXXXXXXXXXXXXXXXXXXXXXXXXXXXXXXXXXXXXXX
XXXXXXXXXXXXXXXXXXXXXXXXXXXXXXXXXXXXXXXXXXXXXXXXXXXXXXXXXXXXXXXXXXXXXX
XXXXXXXXXXXXXXXXXXXXXXXXXXXXXXXXXXXXXXXXXXXXXXXXXXXXXXXXXXXXXXXXXXXXXX
XXXXXXXXXXXXXXXXXXXXXXXXXXXXXXXXXXXXXXXXXXXXXXXXXXXXXXXXXXXXXXXXXXXXXX
XXXXXXXXXXXXXXXXXXXXXXXXXXXXXXXXXXXXXXXXXXXXXXXXXXXXXXXXXXXXXXXXXXXXXX
XXXXXXXXXXXXXXXXXXXXXXXXXXXXXXXXXXXXXXXXXXXXXXXXXXXXXXXXXXXXXXXXXXXXXX
XXXXXXXXXXXXXXXXXXXXXXXXXXXXXXXXXXXXXXXXXXXXXXXXXXXXXXXXXXXXXXXXXXXXXX
XXXXXXXXXXXXXXXXXXXXXXXXXXXXXXXXXXXXXXXXXXXXXXXXXXXXXXXXXXXXXXXXXXXXXX
XXXXXXXXXXXXXXXXXXXXXXXXXXXXXXXXXXXXXXXXXXXXXXXXXXXXXXXXXXXXXXXXXXXXXX
XXXXXXXXXXXXXXXXXXXXXXXXXXXXXXXXXXXXXXXXXXXXXXXXXXXXXXXXXXXXXXXXXXXXXX
XXXXXXXXXXXXXXXXXXXXXXXXXXXXXXXXXAAGTGTCGGACCAATTGATATCTGCGTCATAGXXXXX
XXXXXXXXXXXXXXXXXXXXXXXXXXXXXXXXXXXXXXXXXXXXXXXXXXXXXXXXXXXXXXXXXXXXXX
XXXXXXXXXXXXXXXXXXXXXXXXXXXXXXXXXXXXXXXXXXXXXXXXXXXXXXXXXXXXXXXXXXXXXX
XXXXXXXXXXXXXXXXXXXXXXXXXXXXXXXXXXXXXXXXXXXXXXXXXXXXXXXXXXXXXXXXXXXXXX
XXXXXXXXXXXXXXXXXXXXXXXXXXXXXXXXXXXXXXXXXXXXXXXXXXXXXXXXXXXXXXXXXXXXXX
XXXXXXXXXXXXGAAT
>maker-ChrY-snap-gene-1.17
ATGAGTATTATGCCCTTTTTACAATATAGCGAAACATCGGATAGAATTCTAGGTTTTGAAGACTATGGAG
AGAAAGAACAATATAAAAAAATTTCTGACCACGCCTTGGTATTCATGTTAAGAGGAATACGAAAAAAATT
TAAACAGCCAATTCTCTTTCCTTTTATCAATCGCAAACGTCTACTCCAAATC
>maker-ChrY-exonerate_protein2genome-gene-1.9
ATGAAAAGTCCTAAGATTTGTGTAGCGACAACGTTGGAGTCCTATCTTCGAAGAACTGCACCGCTTAAAG
CAAATTTTCAAAAACTCXXXXXXXXXXXXXXXXXXXXXXXXXXXXXXXXXXXXXXXXXXXXXXXXXXXXX
XXXXXXXXXXXXXXXXXXXXXXXXXXXXXXXXXXXXXXXXXXXXXXXXXXXXXXXXXXXXXXXXXXXXXX
XXXXXC
>maker-ChrY-exonerate_protein2genome-gene-1.10
CTAACGTCAAATAATCCTACGAACAGTTTTGATAATAAATCTGCAAAAACATTTAGGAATGCATCAACAC
AAACTGACGAATGTACTGAAAAGAAATCCCGATGTGAAATAGGTATTCAAGTTGATACTTTTACTGGTAA
CCCTATAAGCAACGTTGTTACAAGTGACAAATCTTTGAATATTTTAACGGGGATTGCAAATTTAAGTCTT
CTACATTTTTTAATTAATGTGCTTTCTAACAAAAAAGTAGATAGTAGAATACATCGCTTAACAATTAAAG
AACGTATTCTATTAGTATTTGTTAAGTTAAAATTAGATTTGACTTACTCAGCATTAGGAGTGTTATTTGG
GAATATCAGTGCTACTACAACAAAAAAATTATTTTGTAATACGGTTCAGATACTTGCAGACATTATGCAA
XXXXXXXXXXXXXXXXXXXXXXXXXXXXXXXXXXXXXXXXXXXXXXXXXXXXXXXXXXXXXXXXXTCAGA
AAGTGCGAATTGTCCTTGATTGTACAGAAATCCCAGTTCAAGTTCCACGAAAGTTATGTTGTCGCATCAA
GTTGTATTCAAATTATAAAAACATCATACATTAA
>maker-ChrY-snap-gene-1.23
AGGCACAGTGATTTGAACATAGCTACTATCTTTAGTAAAAAAAATGATAAAATCACACACAGATAAATTG
AGAATAGCCATGCCAAGXXXXXXXXXXXXXXXXXXXXXXXXXXXXXXXXXXXXXXXXXXXXXXXXXXXXX
XXXXXXXXXXXXXXXXXXXXXXXXXXXXXXXXXXXXXXXXXXXXXXXXXXXXXXXXXXXXXXXXXXXXXX
XXXXXXTCTTTGAGGTCTTTCACTGTTAAATATAACACCATCAGGTGAGTATCCAAACCAAGGATATTTA
XXXXXXXXXXXXXXXXXXXXXXXXXXXXXXXXXXXXXXXXXXXXXXXXXXXXXXXXXGTGTTAAATGCTA
AGTCCTCGTTTTCAAGTCCATGTCTCATTTCTTTCGAATATACATTTTTAGGATGGAAGAAATTTTTGAG
TTTAA
>maker-ChrY-snap-gene-1.18
TTAAACTCAAAAATTTCTTCCATCCTAAAAATGTATATTCGAAAGAAATGAGACATGGACTTGAAAACGA
GGACTTAGCATTTAACACXXXXXXXXXXXXXXXXXXXXXXXXXXXXXXXXXXXXXXXXXXXXXXXXXXXX
XXXXXTAAATATCCTTGGTTTGGATACTCACCTGATGGTGGTGTTATATTTAACAGTGAAAGACCTCAAA
GAXXXXXXXXXXXXXXXXXXXXXXXXXXXXXXXXXXXXXXXXXXXXXXXXXXXXXXXXXXXXXXXXXXXX
XXXXXXXXXXXXXXXXXXXXXXXXXXXXXXXXXXXXXXXXXXXXXXXXXXXXXXXXXXXXXCTTGGCATG
GCTATTCTCAATTTATCTGTGTGTGATTTTATCATTTTTTTTTTACTAAAGATAGTAGCTATGTTCAAAT
CACTGTGCCTT
>maker-ChrY-exonerate_protein2genome-gene-1.16
TTAATCATGTAAATACAATGCATTGCGGATGATAGGTTTACTTAAATTTAXXXXXXXXXXXXXXXXXXXX
XXXXXXXXXXXXXXXXTATCTATTAGATGAACCGGGTAAGCACTTTTAAATATTTTAAAAACCTTAATTC
TAGCGTTAACCCTCTCAATGTGTACACGTGCTGAGGCAATACTTCGGTTCATACAAXXXXXXXXXXXXXX
XXXXXXXXXXXXXXXXXXXXXXXXXXXXXXXXXXXXXXXXXXXXXXXXXXXXXXXXXXXXXXXXXXXXXX
XXXXXXXXXXXXXXXXXXXXXXXXXXXXXXXXXXXXXXXXXXXXXXXXXXXXXXXXXXXXXXXXXXXXXX
XXXXXXXXXXXXXXXXXXXXXXXXXXXXXXXXXXXXXXXXXXXXXXXXXXXXXXXXXXXXXXXXXXXXXX
XXXXXXXXXXXXXXXXXXXXXXXXXXXXXXXXXXXXXXXXXXXXXXXXXXXXXXXXXXXXXXXXXXXXXX
```

```
XXXXXXXXXXXXXXXXXXXXXXXXXXXXXXXXXXXXXXXXXXXXXXXXXXXXXXXXXXXXXXXXXXXXXX
XXXXXXXXXTGTTCATTAATATATTTTCTTTTGTAGGTAAATATATAAAACATTGCATAATGTCTGCAAG
TATCTGAACCGTATTACAAAATAATTTTTTTGTTGTAGTAGCACTGATATTCCCAAATAACACTCCTAAT
GCTGAGTAAGTCAAATCTAATTTTAACTTAACAAATACTAATAGAATACGTTCTTTAATTGTTAAGCGAT
GTATTCTACTATCTACTTTTTTGTTAGAAAGCACATTAAT
>maker-ChrY-snap-gene-1.25
AGGCACAGXXXXXXXXXXXXXXXXXXXXXXXXXXXXXXXXXXXXXXXXXXXXXXXXXXXXXXXXXXXXXX
XXXXXAGCCATGCCAAGXXXXXXXXXXXXXXXXXXXXXXXXXXXXXXXXXXXXXXXXXXXXXXXXXXXXX
XXXXXXXXXXXXXXXXXXXXXXXXXXXXXXXXXXXXXXXXXXXXXXXXXXXXXXXXXXXXXXXXXXXXXX
XXXXXXTCTTTGAGGTCTTTCACTGTTAAATATAACACCATCAGGTGAGTATCCAAACCAAGGATATTTA
XXXXXXXXXXXXXXXXXXXXXXXXXXXXXXXXXXXXXXXXXXXXXXXXXXXXXXXXXGTGTTAAATGTTA
AGTCCTCGTTTTCAAGTCCATGTCTCATTTCTTTCGAATATACATTTTTAGGATGGAAGAAATTTTTGAG
TTTAA
>maker-ChrY-snap-gene-1.20
CGTTGTTTATGTCTGAACGTGGTAAGACAXXXXXXXXXXXXXXXXXXXXXXXXXXXXXXXXXXXXXXXXX
XXXXXXXXXXXXXXXXXXXXXXXXXXXXXXXXXXXTTTGAAGATTTCGATGATTTTGAGTCTGATTTTAT
GCAAGCAGATAAGTCCCATAAACAACATCTTCAGGAAATGGAAATXXXXXXXXXXXXXXXXXXXXXXXXX
XXXXXXXXXXXXXXXXXXXXXXXXXXXXXXXXXXXXXXXXXXXXXXXXXXXXXXXXXXXXXXXXXXXXXX
XXXXXXXXXXXXXXXXXXXXCCTCAAGAATGGACAATTGAGCGGTTAGCTGAAGGCTTTCCTGCTTCACC
CGAAGTTATACAAACAAGTAATGACGATAGTTTTGAAAACAGTCCGTTAGCAGAAGCAAACATTTCTGAA
AGTXXXXXXXXXXXXXXXXXXXXXXXXXXXXXXXXXXXXXXXXXXXXXXXXXGTCAAAACTAGTAGAAA
AGGCAGAAATGGGAAAAGAAATAGATGAGAATGAAAAATCTATAATTACTAAGTTCXXXXXXXXXXXXXX
XXXXXXXXXXXXXXXXXXXXXXXXXXXXXXXXXXXXXXXXXXXXXXXXXXXXXXXXXXXXXXXXXXXXXX
XXXXXXXXCAAAAAAAGTCGAAAAAAGATCTGTCTCATT
>maker-ChrY-snap-gene-1.27
TTAAGGCTGTAGACAGATTCCTTGAATCCATGAACTTAAATCAGCATCATCTTCACAATCTAGCACCATT
TCTTGGGGAGCATCAGCATGTAGAATTTCTTGAGCTACTGTTCTGCCTGTTAGGTAAGCCCCATGGACCG
TTGAATAAAAATTACAGTGGGTATGTTCGCCAGCAAACAGTACCACAGGTTTGGTTTCCTCTTCATCAAG
GTATAGAGGCTGAGCTAAGCATTCAATATCAATCTGTGAAGCTCCTACAGCTATTGCTGTATATGATCCC
CTTGTATAGGGCTGACTGTGCCAACTTGTGGAGTAAATTTTCTTATACCAATTTTTACTTAAATCATTTT
GCCCTTCAGGCTTATCAGAATCCGACTCCCACAATAACAAGACTTCGGTAATACTGGGATGTAAAAACGG
CCTTTCATACTCTAGTAAAATCTTGTCAACAGTTCCAAACAACAACCGTTCAACTGCTTCTTTCTTATAA
TCCGGTAATGGGGGCTGAAATAACGTATCCATATTTTGTTTGAGTACTCCTAGAGGAATCGTGCAGATAA
TCTGATCTGCTTGAAAGATTTTCCCATTATCACAATGTACTTCAACATTAAATAAAAATTTTTCGCAGCT
TGATTCTCGACTACAAGTTTCGTCAACTACAGTTCGATCAGAATCATCTGAGTCATTTCCGTTATCAATA
CTGTTTCTCTTATTGAGATTCCAACGGATTAATTTAACTGGATGTCCAGTAAGTAGATTTTCAGGAGGTA
TAACTTTTAGTAACAGGTTCTCTAATTTAATATGTTCACCAACACTATCTATACCTTCCGGTGGTAAATA
CTGTGATAAGAAATACTCTTCGCAACGTCGTAGAAAXXXXXXXXXXXXXXXXXXXXXXXXXXXXXXXGTC
GAAAATGGTACTTGTTTGCCGTTTTCAGTAGCTGCTATAACTTTGTGAGGTTTCGGCGTTTGCATGATGT
CGACAAGCCCATGCTGCATGGCCAATTCGTAGACGGGGTTGCCCAAGACTCCGTGGATCCAATTAGCGCC
CAATTCCACCGATTCACTGCCGATTTTGATTTGAACTATGCGGCCCCCAACTCGATTACGGGCTTCGAGG
AGTTTGTAGTCGGTGAAGCCGTTTTTTGACAAGTGGTACGCTGCTGATAAGCCCGCCATTCCCGCCCCTA
TTATCAGCACTTTACATTTTGGGGGGTCTTTCTCAGTTGGGGGTTTTTCAGTACTTTCCAT
>maker-ChrY-snap-gene-1.28
CTACGCGTTATAACTACACGAAAAAACCTCAGAAAAACACTCTAGGAACAAAGGAAGAACCTCTTCTATT
GTAACATGTCTGTTTAGTTCCTTGCTGAGACTAGTAACTCCTTTACCTTCAATACCACATGGTACAATAT
GGTCAAACCATTTTAAATCAGTACAACAGTTAAGAGCAAGACCATGTGAGGTCACAAACCGACTTCCATG
CACTCCAATTGCACAAATCCAAACTCCAGTGTAAGAGGACGTTTTTGCCCCAATCCCCATTTTGTGACAC
AATAGTATCACAGTTTTCTCAATTTGAGACACATACCATCTCATXXXXXXXXXXXXXXXXXXXXXXXXXX
XXXXXXXXXXXXXXXXXXXXXXXXXXXXXXXXXXXXXXXXXATCAAGCCGCCCTTCCTGGGGGTAGTCTT
TTGACCGGATTCCAAGCGTGAACACAGGGGGGTGTTCAAGACATAAAAGGGTGTTAGCAAGCTGGGAATT
TTGTTGATGCAAAGTAACAAGATATTTTGGAGAGCCAAACCGTTTTTATAGCTTAAACGGCCCACTTGCC
ACACTTTTATTAGCTTCAACAT
>maker-ChrY-exonerate_est2genome-gene-1.2
AATAATTTGGTTAATTTTCGATTAATTATGTCTGTTGATGAAGAAAAAGACGTAACGGATAAAAAAATTC
GTTTTAAGACACCTGCCCGTGTTTTATTTACAACTGAAAATAAAATTGAGGATGTACCACGAAATGACAA
GGAACCTTCAGAGGTGGXXXXXXXXXXXXXXXXXXXXXXXXXXXXXXXXXXXXXXXXXXXTACAAGTGGAG
TTGACACGTGACTATATTTTGGAGCATGATGAAGAACGATTTTCTGCAAGGAGAGAAAAAGTATATTCGT
TTATGAGAATACCAAGAGAAGTXXXXXXXXXXXXXXXXXXXXXXXXXXXXXXXXXXXXXXXXXXXXXXXX
XXXXXXTTATACTTTTTTACCAATGAGAGTTGTACTAGCAATTTGGGCTCTAATAACTCGACCATTTTCA
CAATGTTTTGGTTCACGTGACAGACGAAAGAAACGGATTTTAACACCAGCTGAAATCTGTGATCTGCTTA
AGGCTGTTATTTTAATTGTTTGXXXXXXXXXXXXXXXXXXXXXXXXXXXTAATATGTTATATCATTTGAT
CAAGAGCCAATCAGTGATTAAATTGTACATTTTCTACAATATGTTAGAAGTTGGAGATAGATTATTTTCT
GCATTTGGACAAGACACTATTGATGCACTTTTCTGGACTGCTACTGAACCCAGAGGGAGAAAAAGGGAAC
ACTTAGGCGTGATACCACACCTTGTTTTTGCTATTGTCTATGTCATACTTCACAGTATATTAGTCCTGCT
TCAAGCCACAACACTTAATGTAGCCATCAATGCAAACAACAAAGCTCTCCTGACCATTATGATGTCTAAT
AATGTAAGTAAAAGTAACGGGTGACTACAAAAATCGAAATTTAAGTAGGTGGATGTAACTTTAAACCAAA
```
