# Supplementary material for: Sex and neo-sex chromosome evolution in beetles
Source: PLoS Genet. 2024 Nov 25;20(11):e1011477. doi: 10.1371/journal.pgen.1011477 (PMC11753715; doi:10.1371/journal.pgen.1011477)
Supplement: Attachment — Submitted filename: 0_Response_reviewers.docx [file pgen.1011477.s019.docx]

Dear Editors,

Please find enclosed a revised version of our manuscript "Sex and neo-sex chromosome evolution in beetles". Below is a point-by point response to each reviewer and the associate editor. We hope that you find our revised version suitable for publication in PLoS Genetics.

Best wishes

Doris Bachtrog

**Response to Editorial Comments**

The manuscript is written clearly and there were no concerns with your overall interpretation of the results. The reviewers had some recommendations to improve the overall clarity of the manuscript. In particular, there were some areas of the methods that were not adequately described. In addition, two reviewers were concerned with the accessibility of your datasets. It appears the resequencing, genome assembly, and RNA-seq data are available under BioProject PRJNA715483. Please make sure all datasets are publicly available here.

*We made sure that the methods are adequately described, and that all datasets are publicly available.*

The reviewers also suggested some additional analyses with the genes on the Y chromosome. The total number of genes on the Y chromosome were only mentioned in Table 1. It would helpful to explore these genes further. In other species, genes on the Y chromosome can be enriched for male specific functions and can have interesting patterns of selection. In T. confusum, are the 24 remaining genes enriched for any particular functions? Are these ancestral genes?  In addition, are any of these genes under any form of selection? 

*We did not include an analysis of the 24 Y genes, as we were not particularly confident in the annotation of the highly repetitive Y chromosome. We now add some supplementary material where we report on function, expression and orthologs of those Y genes. We indeed find that about 1/2 of the annotated Y genes have an ortholog on the X (11 of 24) and may thus represent ancestral genes. However, most of these genes show very low or no expression in male tissue, and thus may not be real functional genes, and many of the annotated Y genes contain (fragments of) repeats. This is discussed in the supplementary Note 1.*

Please be aware that our [data availability policy](http://journals.plos.org/plosgenetics/s/data-availability) requires that all numerical data underlying graphs or summary statistics are included with the submission, and you will need to provide this upon resubmission if not already present. In addition, we do not permit the inclusion of phrases such as "data not shown" or "unpublished results" in manuscripts. All points should be backed up by data provided with the submission.

*All numerical data underlying graphs and summary statistics as well as genome assemblies and annotations are available in a permanent repository on Figshare (*[*https://figshare.com/projects/Sex_chromosome_evolution_in_beetles/157650*](https://figshare.com/projects/Sex_chromosome_evolution_in_beetles/157650)*).*

*There is no “data not shown” or “unpublished results” in our manuscript.*

*During revision, we updated Table 1 since upon submission of the genome assembly NCBI trimmed scaffolds that ended in Ns (a result of a scaffolding script provided with 3d-DNA) and they also identified nontarget sequence on the end of chromosome Chr9_2 that was subsequently removed. These modifications slightly changed the gene number estimate for Chr9_2 and slightly changed the length estimates for all chromosomes. All numbers now match the genome assembly that is deposited at NCBI for Tribolium confusum.*

[*https://www.ncbi.nlm.nih.gov/datasets/genome/GCA_019155225.1/*](https://www.ncbi.nlm.nih.gov/datasets/genome/GCA_019155225.1/)

*Additionally, in response to Reviewer 2, we reran our Repeatmodeler2 and Repeatmasker with newer versions which slightly changed the percentage repetitive estimate shown in Table 1. Although likely imperceptible in Figure 2B, we also replotted to update that figure.*

**Response to Reviewer #1:**

This is a nice study examining the evolutionary history of sex chromosomes in beetles. I really have only minor queries/items of clarity, and one suggestion for complementary data analysis.

1) There doesn't seem to be any mention of the structure and gene composition of the assembled Y, other than its presence in Table 1. This is surprising. What genes are remaining on the Y, are there spatial patterns of loss, and are there interesting patterns of gene retention/loss by gene function?

*See response to the Associate Editor above. We now include Supplementary Note 1 that provides some analysis of the annotated Y genes (Lines 117-118). However, we caution that we are not particularly confident in the annotation of the highly repeat-rich Y chromosome, and whether most of the predicted genes are indeed real genes or simply fragments of repeats.*

2) I would recommend the authors try GeneSpace as an alternative/complement to identify and visualize syntenic orthologues across these genomes, I think it can help provide some clearer patterns of visualization across species. (<https://github.com/jtlovell/GENESPACE>). I find the decisions around the "Stevens element' designation to be semi-arbitrary from the synteny plots shown, but perhaps it would be more convincing with Genespace plots, and/or some additional justification of how these were decided on in the methods. But great tribute to Nettie Stevens with the naming!

*Thank you for the suggestion! We have now included an analysis using GENESPACE and find the results to be qualitatively similar to our previous analyses, but the plots are clearer. We have updated Figure 4 and added text (Lines 426-508) and placed the old Figure 4B in the supplement since it still aids in interpretation.*

**Response to Reviewer #2:**

This is the first study to comprehensively reconstruct sex chromosomes, their characteristics, and evolution in detail in a large number of beetle species. Beetles are a very specious group with potentially numerous independent origins of new sex chromosomes and so represent a very valuable model to study the origin and evolution of sex chromosomes and sex determination. However, as yet they have received limited attention in this regard. This elegant study generates genomic and expression data for several species and combines this will publicly available data across eleven species. They identify significant patterns of sex chromosome evolution and open up new avenues for future research. The paper is well written and easy to follow for a broad audience and the figures are well presented. I have only minor comments.

Methods are lacking detail in places. I would expect to see a Supplementary Methods section for these types of analyses.

*We have extended the methods is several places to give the readers more detail (see Lines 323, 338, 340, 347, 348, 401-405, 407-413, 426-508).*

L268 Can the authors include information on the thresholds used to identify non-target sequences.

*We have added details about our BLAST search of small contigs and how we determined which contigs to retain in the final genome assembly (line 323). It is also worth noting that the genome assembly was passed through NCBIs contamination screen by NCBI when the genome was submitted and published online.*

L282 - 285 Further details on the thresholds used in the 3d-DNA pipeline are necessary

*We have now included text on how 3d-DNA was run (lines 337-341). We chose not to allow 3d-DNA to do any rounds of misjoin correction based on our experiences with other genome assemblies (Bracewell et al. 2019, Bracewell et al. 2020).*

L296 How were SNAP and Augustus run
*We have now included additional information on Augustus and SNAP settings used in our MAKER annotation (lines 401-404).*

L319 Where did the D. brevicomis genome come from? Is it publicly available or assembled as a part of this paper?

*The assembly comes from Bracewell et al. 2018 and from the Illumina reads that are published under Accession SRX4297965. The assembly is highly fragmented and of somewhat limited use but was deposited on Figshare (below) along with other useful files. We have added this figshare link to the data accessibility statement.*

<https://figshare.com/projects/Sex_chromosome_evolution_in_beetles/157650>

Given the significant enrichment in repetitive elements, it would be helpful for readers to break this down by type of element in a new table.
*We have now added a table that includes this information (Table 2).*

The authors have made the genome assembly and raw reads available but not the RNA-seq data they generated for T. confusum or the resequencing data for D. brevicomis.

*All data is now available under the Bioproject that is referenced in the paper.*

[*https://www.ncbi.nlm.nih.gov/bioproject/?term=PRJNA715483*](https://www.ncbi.nlm.nih.gov/bioproject/?term=PRJNA715483)

L12 Missing comma after Tribolium confusum

*Fixed.*

L46 Unnecessary comma after high conserved

*Fixed.*

L58 Should be evolving

*Fixed.*

L60, L206 Numbered references Charlesworth & Charlesworth 2000 Bachtrog 2013 & WHITTLE et al. 2020

*We fixed the references.*

L63, 65 Need references to support these statements

*We added citations to these statements.*

L311 Typo should be and

*Fixed.*

L291 Typo should be characterize

*Fixed.*

L115 Should Figure 2C be referenced here?

*We have removed the Fig. 2C reference.*

**Response to Reviewer #3:**

The authors provide the most complete genomically informed examination of sex chromosome evolution in beetles to date. I believe this is an important contribution that will be of broad interest. I think there are a few things that could be added or adjusted to make the article even better prior to publication.

Line 50 This is more of a question for the authors. Each time I read places where you say, "chromosomal sex determination is conserved across beetles," I kept asking myself whether haplodiploidy is considered a form of chromosomal sex determination. I wonder if, in the introduction, it might be good to provide some basic information on the proportions of species with XY, XO, HD, and parthenogenetic systems. I think this would give a broader perspective and context for the study.

*We now include the following paragraph (lines 41-45). “In particular, information on sex determination is available for over 4500 beetle species, and 93% of species reported show genetic sex determination (17% are X0 and 75% have XY sex chromosomes). Haplodiploidy and paternal genome elimination is found in only 10 species (0.2%), and parthenogenesis has been found for 7% of the species investigated (2).”*

Line 57 It might be worth mentioning in the introduction that you are including species that have non-pairing sex chromosomes and pairing sex chromosomes and variation in this characteristic across beetles. The fact that you mention the yp (distant pairing parachute Y chromosome) notation for some sex chromosomes later (line 156) makes me feel more strongly that you should explain variation in pairing and whether you think this impacts the course of sex chromosome evolution in your Adephaga species vs the Polyphaga species that are dominated by distant pairing sex chromosomes.

*We include the following paragraph in the introduction (lines 47-51). “Beetle sex chromosome karyotypes are typically inferred from testis squashes, and often reported as XY, X0, and Xy+ (2, 7). XY sex chromosomes typically undergo synapsis during meiosis and can recombine in their pseudoautosomal region in males (but note that achiasmatic male meiosis has evolved in some groups (2, 7)). Xy+ sex chromosomes, on the other hand, pair at a distance with no synapsis and no opportunity for recombination during male meiosis (2, 7).”*

Line 60 Citations are mixed in number format and author year
*We fixed all the citations throughout the manuscript.*

Line 94 The statement that the NeoY will lose all homology to the Neo X seems to be overly strong. Many Y chromosomes retain some subset of genes that are essential and have gametologs on the X. This seems to be too strong of a blanket statement.
*We agree. We have reworded it to ‘nearly all’*

Line 309 is this new assembly deposited in a repository?

*Yes, the reassembly and new annotation is below. We have also added additional useful files recommended by other reviewers.*

<https://figshare.com/projects/Sex_chromosome_evolution_in_beetles/157650>
